# Tylophorine Analogs Allosterically Regulates Heat Shock Cognate Protein 70 And Inhibits Hepatitis C Virus Replication

**DOI:** 10.1038/s41598-017-08815-z

**Published:** 2017-08-30

**Authors:** Ying Wang, Sangwon Lee, Ya Ha, Wing Lam, Shao-Ru Chen, Ginger E. Dutschman, Elizabeth A. Gullen, Susan P. Grill, Yao Cheng, Alois Fürstner, Samson Francis, David C. Baker, Xiaoming Yang, Kuo-Hsiung Lee, Yung-Chi Cheng

**Affiliations:** 1 Department of Pharmacology, Yale University School of Medicine, New Haven, CT 06520 USA; 2 Institute of Chinese Medical Sciences and State Key Laboratory of Quality Research in Chinese Medicine, University of Macau, Macau, SAR China; 3 Max-Planck-Institut für Kohlenforschung, 45470 Mülheim/Ruhr, Germany; 4 Department of Chemistry, The University of Tennessee, Knoxville, TN 37996 USA; 5 Natural Products Research Laboratories, Eshelman School of Pharmacy, University of North Carolina, Chapel Hill, NC 27599 USA; 6 Chinese Medicine Research and Development Center, China Medical University and Hospital, Taichung, Taiwan

## Abstract

Tylophorine analogs have been shown to exhibit diverse activities against cancer, inflammation, arthritis, and lupus *in vivo*. In this study, we demonstrated that two tylophorine analogs, DCB-3503 and *rac*-cryptopleurine, exhibit potent inhibitory activity against hepatitis C virus (HCV) replication in genotype 1b Con 1 isolate. The inhibition of HCV replication is at least partially mediated through cellular heat shock cognate protein 70 (Hsc70). Hsc70 associates with the HCV replication complex by primarily binding to the poly U/UC motifs in HCV RNA. The interaction of DCB-3503 and *rac*-cryptopleurine with Hsc70 promotes the ATP hydrolysis activity of Hsc70 in the presence of the 3′ poly U/UC motif of HCV RNA. Regulating the ATPase activity of Hsc70 may be one of the mechanisms by which tylophorine analogs inhibit HCV replication. This study demonstrates the novel anti-HCV activity of tylophorine analogs. Our results also highlight the importance of Hsc70 in HCV replication.

## Introduction

Tylophorine analogs are a class of natural products first isolated from the plant *Tylophora Indica* ^1^. *Tylophora Indica* and *Tylophora Ovata* were traditionally used for the treatment of asthma in India^2^ and arthritis in China^3^. This group of analogs has also shown broad activities against cancer^4, 5^, inflammation^5, 6^, arthritis^3^, and lupus^7^ *in vivo*. Tylophorine analogs have been shown to reduce the rate of elongation of polypeptide chain to inhibit global protein synthesis^8^, which has a more profound effect on cellular regulatory proteins with short half-lives^8^. No other known anticancer drug or protein synthesis inhibitors exhibits the same novel activity. Only vascular endothelial growth factor receptor 2 (VEGFR2) has been identified for the antiangiogenic activity of tylophorine^9^. In addition, we recently identified that tylophorine analog DCB-3503 regulated translation of cyclin D1 through allosteric regulation of heat shock cognate protein 70 (Hsc70)^10^.

Hepatitis C virus (HCV) infects approximately 3% of the world’s population and is one of the top reasons for the development of liver cirrhosis and hepatocellular carcinoma^11^. The nonstructural (NS) proteins are responsible for replication of HCV RNA and virus particle assembly and are primary antiviral targets^12, 13^. The initial standard of care for HCV infection is peginterferon alpha combined with ribavirin for 48 weeks for HCV genotype 1, or 24 weeks for HCV genotypes 2 or 3. Peginterferon treatment showed a sustained viral response (SVR) rate of less than 50% HCV genotype 1 infection, the most prevalent strain in Europe and North America^11^. The recently developed direct-acting antivirals (DAAs) targeting against NS proteins significantly improved SVRs to as high as 99% and broadened the spectrum of patients to all genotypes as well as HCV with cirrhosis patients^14–16^. However, the high cost of current treatment restricts broad patient access, especially to the DAAs. In addition, targeting host factors by small molecules could illustrate the important functions of host factors involved in viral replication and pathogenesis^17^.

Tylophorine analogs inhibit selected set of protein syntheses by inhibiting the elongation step^8^. Both HCV replication and protein synthesis take place on the endoplasmic reticulum (ER). Therefore, we explored whether tylophorine analogs could inhibit replication of HCV. We hypothesized that DCB-3503 inhibits elongation by interacting with host factors; thus we aimed to identify cellular target(s) of the tylophorine analogs regulating translation and viral replication. Using biotinylated-DCB-3503^4^ and -r*ac*-cryptopleurine^18^, we identified heat shock cognate protein 70 (Hsc70) as one of the cellular targets of tylophorine analogs. Hsc70 is a constitutively expressed heat shock protein family member with an ATPase N-terminal nucleotide-binding domain (NBD) and a C-terminal substrate-binding domain (SBD)^19^. The ATPase activity of Hsc70 could be regulated by small-molecule inhibitors and/or cochaperone proteins^20^.

In this study, we demonstrated that DCB-3503 and *rac*-cryptopleurine potently inhibit HCV replication. They both exhibit anti-HCV activity by promoting the ATP hydrolysis activity of Hsc70 through binding to the 3′ poly U/UC motif of HCV RNA. This allosteric regulation of Hsc70 function alters the interaction between Hsc70 and its associated HCV protein and RNA, and also perturbs the homeostasis of the HCV replication complex, thus inhibiting translation of HCV RNA.

## Results

### Tylophorine analogs exhibit anti-HCV activity in HCV genotype 1b replicon cells

**Figure 1:**
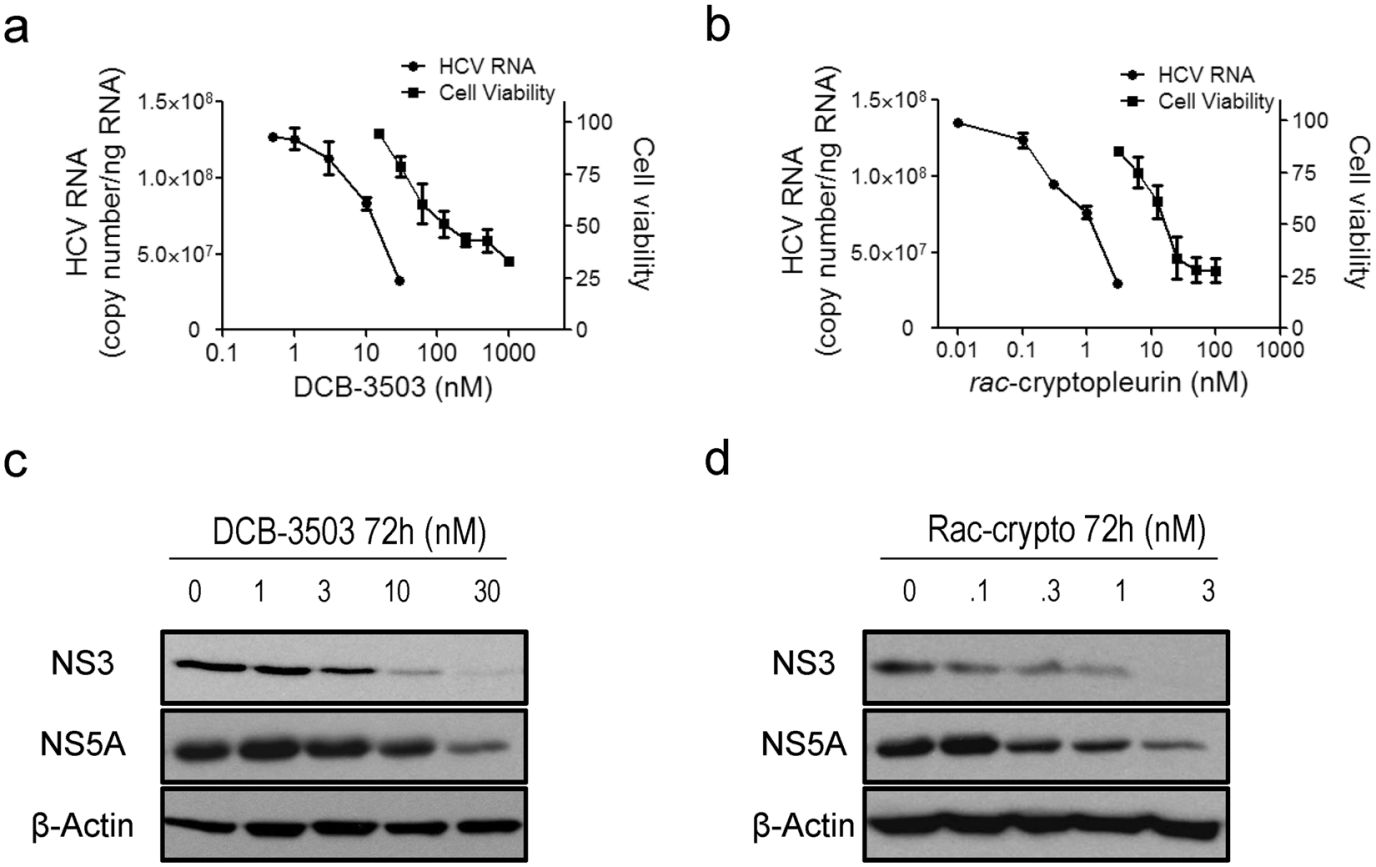
DCB-3503 and *rac*-cryptopleurine exhibit anti-HCV activity in HCV genotype 1b (Con1) replicon cells. The level of HCV RNA and cell viability after treatment with various concentrations of (**a**) DCB-3503 or (**b**) *rac*-cryptopleurine. The expression level of HCV RNA was analyzed by real-time PCR and normalized to that of β-actin. Expression of NS3 and NS5A in the (**c**) DCB-3503 and (**d**) *rac*-cryptopleurine-treated HCV replicon cells were analyzed by Western blot. β-Actin served as the internal loading control. The results are representative of at least three independent experiments, error bars represent standard deviations.

We previously demonstrated that DCB-3503 and its analogs inhibit protein synthesis^8, 21^. Protein synthesis is one of the most critical steps in HCV replication^11^; therefore, we examined the anti-HCV activity of the tylophorine analogs, DCB-3503 and *rac*-cryptopleurine^8, 21–23^ in Huh-luc/neo-ET cells harboring the HCV genotype 1b Con 1 isolate. Treatment with DCB-3503 and *rac*-cryptopleurine decreased HCV RNA levels (Fig. 1a and b) and the expression of NS3 and NS5A proteins in a dose-dependent manner (Fig. 1c and d). No resistant clones could be isolated after selection with a 10-fold EC_50_ concentration of DCB-3503 (300 nM) and *rac*-cryptopleurine (30 nM) for three weeks (Figure S1a). While resistant clones emerged under the selection of a 10-fold EC_50_ concentration of *meso*-tetrakis-porphyrin compound **6** (200 nM), a potent HCV inhibitor developed in our laboratory that targeta viral protease (Figure S1a)^24^.

### Tylophorine analog specifically interacts with Hsc70

**Figure 2:**
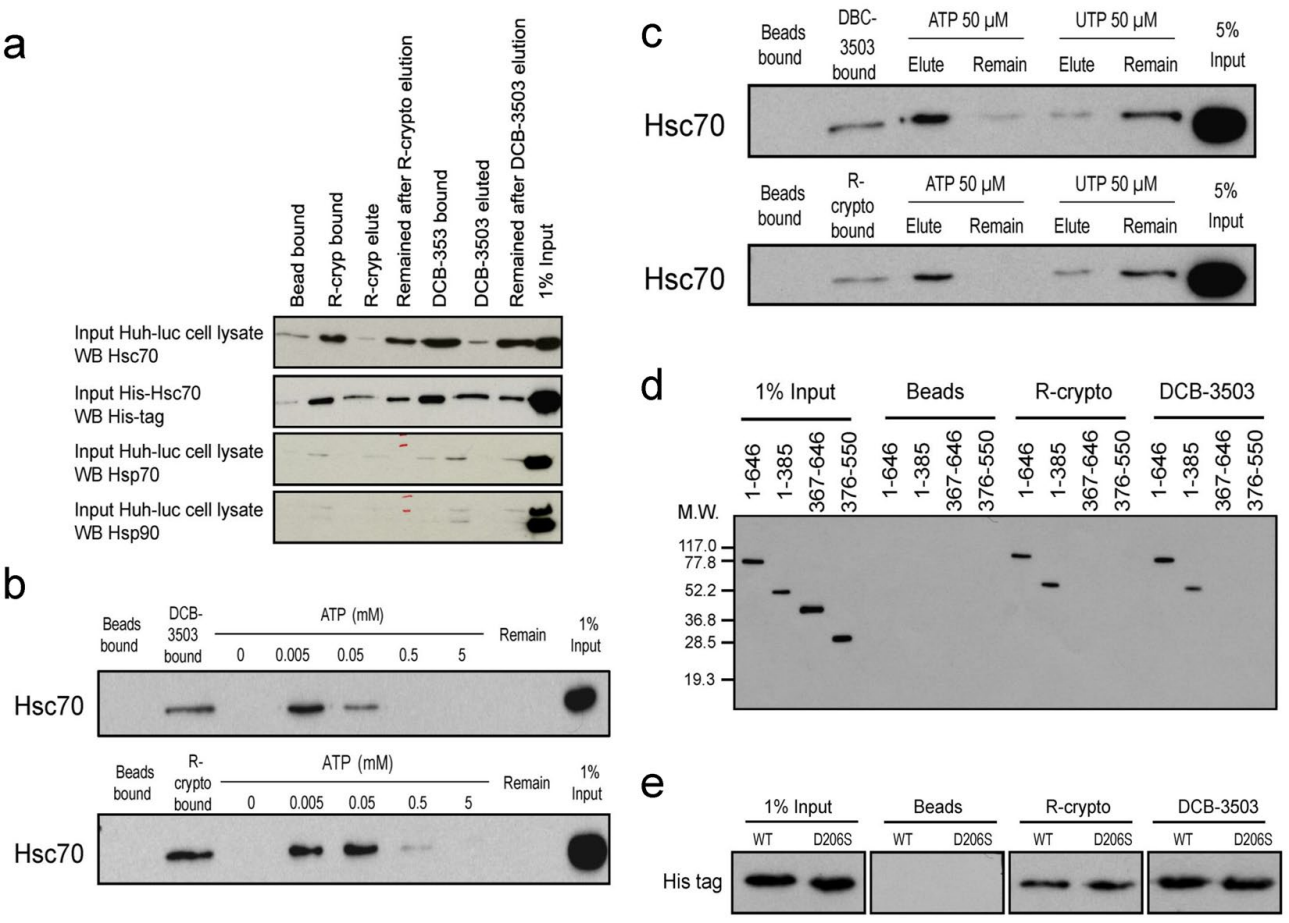
Tylophorine analogs interact with Hsc70. (**a**) Hsc70, but not Hsp70 or Hsp90, bound specifically to *rac*-cryptopleurine or DCB-3503 affinity resin. (**b**) Bound recombinant Hsc70 was eluted with increasing concentrations of ATP. The Hsc70 in each fraction was resolved by Western blot. (**c**) Bound recombinant Hsc70 was eluted with 50 µM ATP or UTP. Hsc70 in each fraction was resolved by Western blot. (**d**) His-tagged different fragments of Hsc70 were incubated with streptavidin-agarose beads coated with biotinylated-*rac*-cryptopleurine or -DCB-3503. Bound proteins were immunoblotted with anti-His tag antibody. (**e**) His-tagged Hsc70 protein harboring D206S mutation was incubated with streptavidin-agarose beads pre-bound to biotinylated-*rac*-cryptopleurine or -DCB-3503. Bound proteins were immunoblotted with anti-His tag antibody. Results are representative of at least three independent experiments.

Since we could not isolate resistant clones to DCB-3503 or *rac*-cryptopleurine, we hypothesized that tylophorine analogs may inhibit viral replication by affecting host factors. Biotinylated DCB-3503 and *rac*-cryptopleurine were synthesized to determine the molecular target(s) of the tylophorine analogs. The tethered biotin group was connected to the 3-position on the tylophorine backbone as directed by our structure–activity relationship study^8, 10, 21–23^ (Figure S1b). The anti-HCV activity and cytotoxicity of both parental compounds and biotinylated derivatives are shown in Table S3. The flow chart of affinity purification was shown in Figure S1c. The proteins bound to biotinylated-DCB-3503 and -*rac*-cryptopleurine were separated by SDS-PAGE and visualized by silver staining. Proteins that specifically eluted by *rac*-cryptopleurine were identified by LTQ Orbitrap mass spectrometry^10^. Western blot analysis results revealed that biotinylated DCB-3503 and *rac*-cryptopleurine bound to Hsc70 when Huh-luc/neo-ET cell lysate or recombinant polyhistidine (His)-tagged Hsc70 was run through the compound matrix column (Fig. 2a). Hsp70 or Hsp90 did not bind to the affinity matrix under the same conditions (Fig. 2a). Increasing concentration of ATP (Fig. 2b) but not UTP (Fig. 2c) eluted the compound-bound Hsc70. The binding was also dependent on ionic strength: 0.4 M NaCl eluted the bound Hsc70 from the affinity matrix (Figure S1d). Western blot results showed that both DCB-3503 and *rac*-cryptopleurine bound to the NBD of recombinant Hsc70 (residue 1–385) (Fig. 2d). The binding affinity of DCB-3503 and *rac*-cryptopleurine to Hsc70 did not change when a D206S mutation was introduced into the ATPase pocket in the NBD of Hsc70 (Fig. 2e)^25^. In order to determine antibody specificity, we expressed and purified recombinant Hsp70. Western blot results indicated that the Hsc70 antibody we used recognized only recombinant Hsc70 but not Hsp70 (Figure S1e). Isothermal titration calorimetry (ITC) revealed that DCB-3503 and *rac*-cryptopleurine showed similar binding affinities to the full length (FL) and the NBD of Hsc70 (Table 1 and Figure S1f **)**. The protein level of Hsc70 remained unchanged throughout the treatment with DCB-3503 or *rac*-cryptopleurine for up to 72 hours (Figure S1g).

**Table 1:** Thermodynamic parameters obtained from the ITC measurement of the binding between tylophorine analogs and Hsc70.

Compound	Protein	n	K_d_ (M)	ΔH (kCal/mol)	ΔS (cal/(mol·K))
DCB-3503	Hsc70 FL	0.41	2.8 ± 0.5	3079	35.7
DCB-3503	Hsc70 NBD	0.49	4.4 ± 0.9	5135	41.7
*rac*-Cryptopleurine	Hsc70 FL	0.57	3.1 ± 0.33	2019	32.0
*rac*-Cryptopleurine	Hsc70 NBD	1.06	2.7 ± 0.48	1905	31.9

### Hsc70 is involved in the anti-HCV activity of DCB-3503

**Figure 3:**
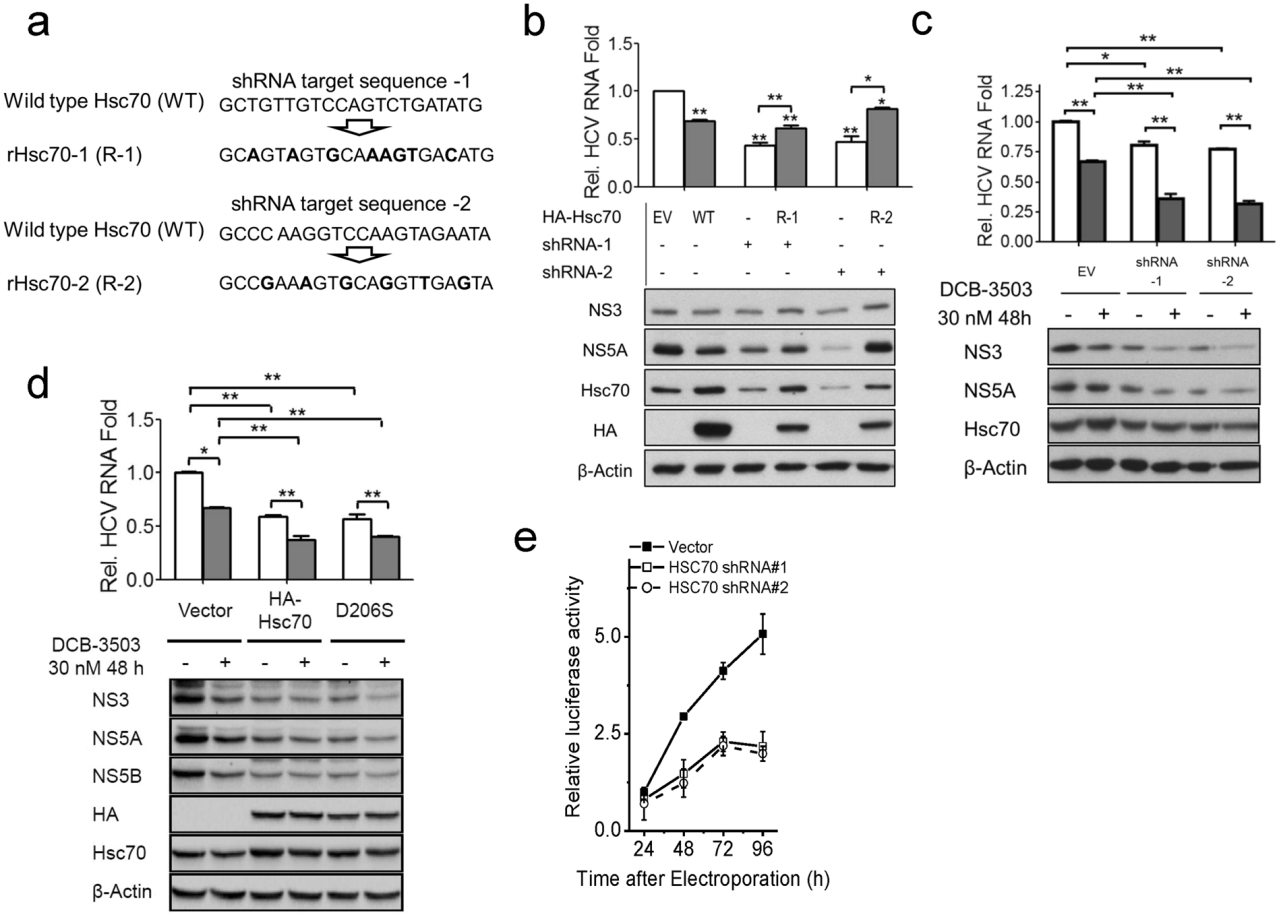
Tylophorine analogs exhibit anti-HCV activity via interaction with Hsc70 in HCV genotype 1b (Con1) replicon cells. (**a**) The shRNA-resistant Hsc70 with nucleotide substitution in the 21-mer targeting sequences were constructed without changing amino acids (rHsc70-1, rHsc70-2). (**b**) Huh-luc/neo-ET cells were transiently transfected in a six-well plate with 400 ng HA-Hsc70 plasmid (WT, R-1, and R-2), alone or in combination with either Hsc70 shRNAs or empty vector (EV), for 36 hours. Levels of HCV RNA, NS3, NS5A, Hsc70, and HA-Hsc70 were analyzed by real-time PCR and Western blot, respectively. β-Actin served as the internal loading control. (**c**) Huh-luc/neo-ET cells were transiently transfected in a six-well plate with 400 ng Hsc70 shRNA or empty vector (EV). The cells were either treated with 30 nM DCB-3503 or untreated 24 hours after transfection. HCV RNA and expression of Hsc70 were examined by Real-Time PCR and Western blot, respectively. The numbers under the bands indicate normalized relative band intensity. (**d**) Huh-luc/neo-ET cells were transiently transfected in a six-well plate with 1 μg WT HA-Hsc70, D206S mutant HA-Hsc70 plasmid, or EV. DCB-3503 was added 24 hours after transfection and treated for another 48 hours. HCV RNA was examined by Real-Time PCR. The expression levels of NS3, NS5A, NS5B, HA tag, and Hsc70 were examined by Western blot. β-Actin served as an internal loading control. (**e**) Transient transfection of HCV RNA into Hsc70 knocked-down HLN-cure cells. 400,000 HLN-cure cells transfected with Hsc70 shRNAs or empty vector 72 hours earlier were transiently transfected with HCV RNA by electroporation. HCV replication was monitored by luciferase assay at 24, 48, 72, and 96 hours post-electroporation. The luciferase activity 24 hours post-electroporation in empty vector transfected HLNC cells was set as 1-fold. The expression levels of Hsc70 before and after electroporation are shown in Figure S2e. Results are representative of at least three independent experiments. Error bars represent standard deviations from three independent experiments. ^#^ *p* < 0.1, *p < 0.05, **p < 0.01.

We constructed an Hsc70 overexpression plasmid with silent mutation on the shRNA targeting sites (rHsc70-1, and rHsc70-2, Fig. 3a) to determine the off-target effect of shRNA. Down-regulation of Hsc70 with shRNA reduced HCV RNA replication and suppressed expression of NS3 and NS5A (lanes 3 and 5 in Fig. 3b). The RNAi phenotype was partially rescued by over-expressing HA-rHsc70s (Fig. 3b), suggesting that suppression of Hsc70 was responsible for the down-regulation of HCV replication. Transfection of wild type (WT) HA-tagged Hsc70 also inhibited HCV RNA replication (Fig. 3b, quantitative results of protein expression are shown in Figure S2a). shRNA knockdown of Hsc70 for 48 hours enhanced the anti-HCV activity of DCB-3503 as reflected by the resultant decrease in HCV RNA, NS3, and NS5A levels (Fig. 3c, quantitative results of protein expression were shown in Figure S2b). Reduced expression of Hsc70 by shRNAs did not significantly affect cell growth (Figure S2c). Complete knockout of Hsc70 with combination of two shRNAs is lethal to cells; thus, a more significant anti-HCV effect could not be demonstrated. Overexpression of either WT or D206S mutant HA-tagged Hsc70 inhibited expression levels of HCV RNA and NS proteins, and further enhanced the anti-HCV effect of DCB-3503 (Fig. 3d, quantitative results of protein expression are shown in Figure S2d) without changing the cell growth curve (Figure S2e). Treatment with DCB-3503 inhibited translation of HCV *in vitro* (Figure S2f), and pretreatment of HLN-cured cells with 30 nM DCB-3503 for 24 hours reduced the cells’ ability to support HCV replication by about 2-fold (Figure S2g). Furthermore, the replication rate of HCV RNA transfected into HLN-cured cells with reduced Hsc70 expression by shRNA was about 3-fold slower than those HLC-cured cells without Hsc70 shRNA treatment (Fig. 3e). The Hsc70 expression levels are shown in Figure S2h. Alteration of expression of Hsc70 is related to HCV RNA replication.

### Hsc70 is associated with the HCV replication complex

**Figure 4:**
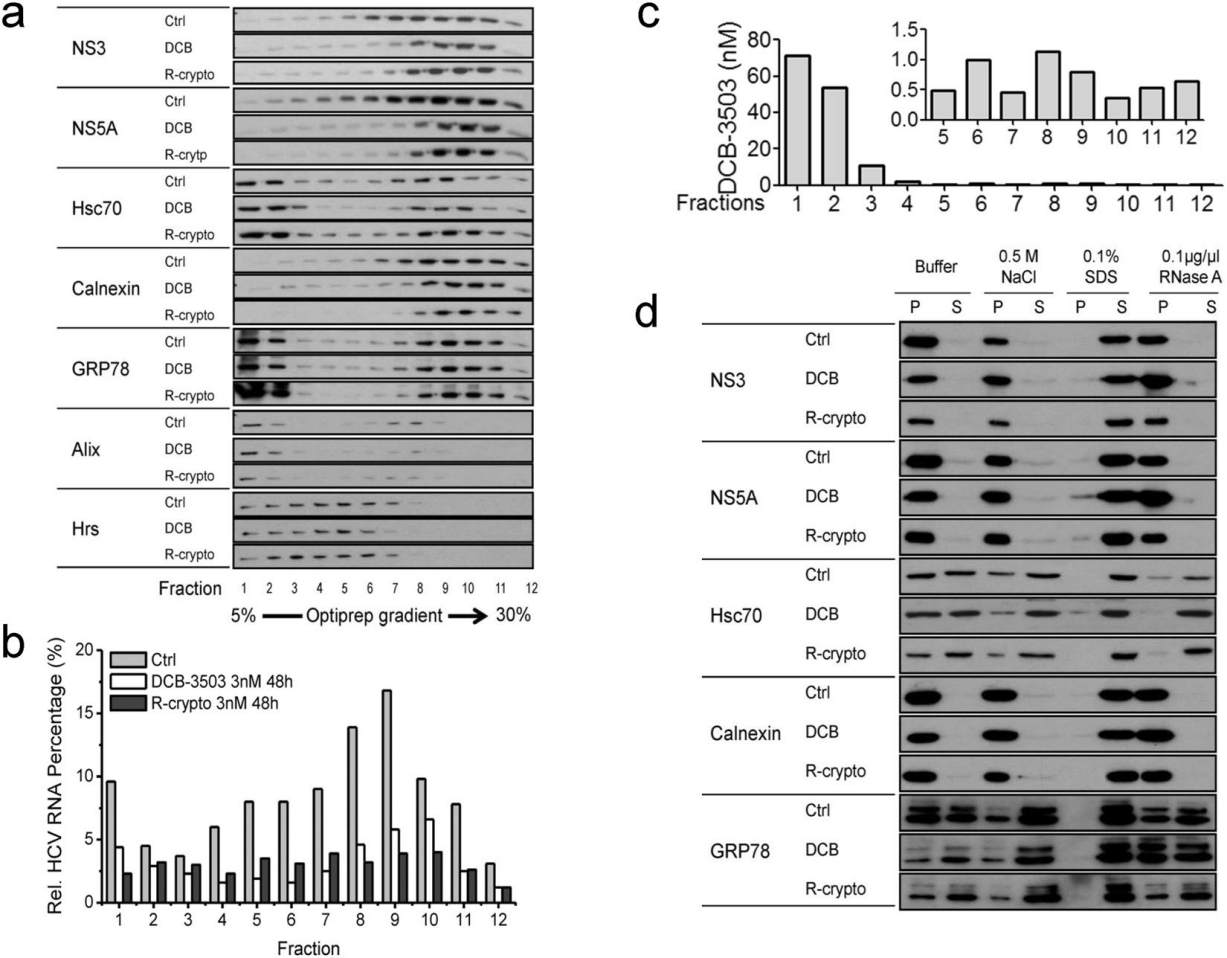
Hsc70 associates with HCV replicon complex. (**a**) Partition of NS3, NS5A, Hsc70, Calnexin, GRP78, Alix, and Hrs from fractions obtained from control Huh-luc/neo-ET cells and cells treated with either 30 nM DCB-3503 (DCB) or 3 nM *rac*-cryptopleurine (R-crypto) for 48 hours. (**b**) The relative HCV RNA content in fractions obtained in (**a**) was analyzed by Real-Time PCR. (**c**) The concentration of DCB-3503 in each fraction was analyzed by LC–MS/MS. The insert shows the zoomed-in results of fractions 5–12. (**d**) Cytosolic fractions of untreated Huh-luc/neo-ET cells and cells treated with 30 nM DCB-3503 or 3 nM *rac*-cryptopleurine for 48 hours were treated with 0.5 M NaCl, 0.1% SDS, or 0.1 μg/μl RNase A for 2 hours before being subjected to centrifugation to isolate CRC. The presence of NS3, NS5A, Hsc70, Calnexin, and GRP78 were analyzed by Western blot. P and S denote the pellet and the supernatant of centrifuged samples, respectively. Results are representative of at least three independent experiments.

Hsc70 exhibits its function via association with different cellular complexes^26^. We thus examined whether Hsc70 is associated with the HCV replication complex. Post-nuclear supernatant of untreated, DCB-3503-treated or *rac*-cryptopleurine-treated Huh-luc/neo-ET cells were fractionated on continuous optiprep iodixanol density gradients (Figs 4a–c, S2i). The optiprep density gradients separated membrane vesicles (Alix and Hrs as markers in fractions 1–6, and GRP 78 as marker in fractions 1–2), and endoplasmic reticulum (ER) (GRP78 and Calenxin as markers in fractions 7–11) (Fig. 4a). In untreated control cells, HCV RNA, NS3, and NS5A exhibited the same sedimentation with the membrane vesicles and ER in fractions 5–11 (Fig. 4a and b), a result consistent with the density reported earlier^27^. In cells treated with DCB-3503 or *rac*-cryptopleurine; however, HCV RNA, NS3, and NS5A agglomerated with the ER in fractions 8–11 (Fig. 4a and b). Hsc70 showed the same sedimentation shift with the membrane vesicles (fractions 1–3) and ER (fractions 7–11) in both untreated control and drug-treated cells (Fig. 4a). DCB-3503 accumulated in the first two fractions with 60–80 nM concentration where Hsc70 was primarily located (Fig. 4c). In addition, DCB-3503 was found in fractions 3–12 at around 1 nM concentration (Fig. 4c). The amount of *rac*-cryptopleurine in the fractions was too low to detect with LC/MS/MS. The cosedimentation of Hsc70 with HCV NS3, NS5A, and ER marker Calnexin was disrupted when the cytoplasmic fraction was treated with 0.5 M NaCl or RNase A before centrifugation to isolate crude HCV replicon (Fig. 4d). All the examined proteins were found only in the supernatant fraction with addition of 0.1% SDS (Fig. 4d). NS3 and NS5A remained in the membrane-rich pellet fraction when ionic strength was changed with NaCl, or when the RNA was depleted with RNase treatment (Fig. 4d). However, addition of 0.5 M NaCl or 0.1 μg/μl of RNase released Hsc70 from the membrane-rich pellet fraction to the supernatant (Fig. 4d). Addition of 0.1% SDS totally disrupted the membrane structure and released NS3, NS5A, and Hsc70 to the supernatant (Fig. 4d).

### Treatment with DCB-3503 promotes binding of Hsc70 to HCV RNA

**Figure 5:**
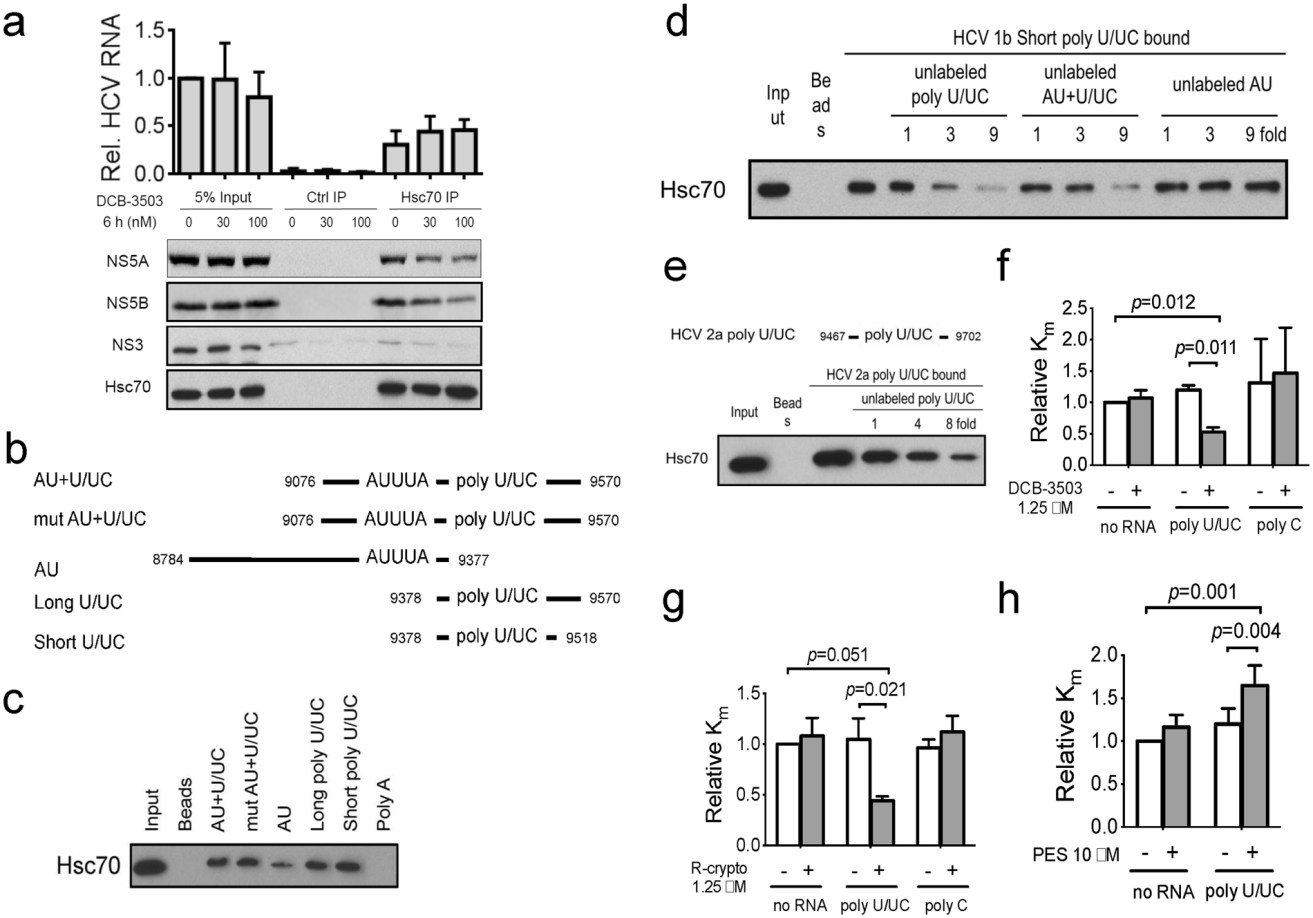
Tylophorine analogs stimulated the ATP hydrolysis function of Hsc70 modulating its association with HCV RNA. (**a**) Hsc70-associated HCV RNA and protein was immunoprecipitated with Hsc70 antibody. The level of HCV RNA was examined by real-time PCR. Protein levels of NS3, NS5A, NS5B, and Hsc70 were analyzed by Western blot. (**b**) The schematic graph showed the RNA sequences used in Fig. 5c,d. Nucleotide positions correspond to the HCV genotype 1b complete genome isolate Con1 (GI 5420376). (**c**) Either streptavidin beads alone or *in vitro* transcribed biotinylated RNA with the sequences shown in (**b**) were mixed with purified Hsc70 protein. RNA-bound Hsc70 was detected by Western blot analysis. (**d**) Unlabeled RNA was used to compete the binding with biotinylated short poly U/UC RNA for binding to Hsc70. (**e**) The schematic graph shows the poly U/UC region of HCV genotype 2a (JFH-1, GI 13122261). The binding assay was done as shown in (**d**). Results of (**a**)–(**e**) are representative from at least three independent experiments. Effect of (f) DCB-3503, (**g**) *rac*-cryptopleurine, or (**h**) 2-phenylethanesulfonamide (PES) on the *K*_m_ of Hsc70 for ATP binding in the presence or absence of short poly U/UC or poly C RNA. The concentration of generated ADP was analyzed and calculated based on the area under the curve (AUC). The *K*_m_ and *V*_max_ values were calculated by fitting the data to the Michaelis–Menten kinetics equation. Error bars represent standard deviations from three independent experiments. Results are representative of at least three independent experiments and presented as mean ± SD. *P* values are labeled on the graph.

Given the result of the co-sedimentation of Hsc70 with HCV NS proteins and RNA, we further examined the association of Hsc70 with HCV replication complex. Real-time PCR results showed that more than 30% of HCV RNA was present in the Hsc70 immunoprecipitated complex (Fig. 5a). The level of HCV RNA in the input and Hsc70-associated complex did not change significantly after treatment with up to100 nM DCB-3503 for 6 hours. However, the amount of Hsc70-associated NS5A and NS5B were reduced with treatment of 30 nM and 100 nM of DCB-3503 for 6 hours (Fig. 5a). NS3/4 A, NS4B, or host RNAs (β-actin and survivin) did not bind to Hsc70 under the same conditions (Figs 5a and S3a,b). RNase A treatment decreased the amount of NS5A and NS5B associated with Hsc70 (Figure S3c). We then analyzed the association between NS5B and Hsc70 and between HCV RNA and NS5B using a co-immunoprecipitation method. DCB-3503 treatment reduced the amount of Hsc70 bound to HA-tagged NS5B when immunoprecipitated with HA antibody (Figure S3d). The association of NS5B and Hsc70 to biotinylated HCV RNA was also decreased with addition of DCB-3503 (Figure S3e). Hsc70 can bind to the AU-rich elements in the 3′ untranslated region of Bim mRNA^28^. Thus, we generated constructs with different specific motifs of the 3′ nontranslated region (3′ NTR) of HCV genotype 1b RNA to determine the Hsc70 binding RNA sequence (Fig. 5b)^29^. Hsc70 bound to all the biotinylated RNA fragments containing AUUUA and poly U/UC sequences (Fig. 5c). Biotinylated RNA containing only the AUUUA sequence showed relatively weak association with Hsc70 compared with poly U/UC RNA (Fig. 5d). Hsc70 was not associated with Poly A RNA under the same conditions (Fig. 5c). The association of short poly U/UC RNA with Hsc70 was comparable to that of other longer RNA fragments (Fig. 5c), which suggests that the poly U/UC sequence is essential but not necessarily sufficient for binding to Hsc70. The poly U/UC RNA below refers to the short form of HCV genotype 1b. Poly U/UC RNA did not bind to fragments of Hsc70 (Figure S3f) or Hsp70 (Figure S3g) under the same conditions. Addition of increasing concentrations of unlabeled poly U/UC or AU + UC RNA competed with the association of biotinylated poly U/UC RNA to Hsc70 in a dose dependent manner (Fig. 5d). As much as 9-fold of unlabeled AUUUA RNA could not compete with poly U/UC binding with the same efficiency (last three lanes, Fig. 5d). Deletion of the poly U/UC region in the 3′ NTR could not support HCV replication (Figure S3h). Since the poly U/UC region in the 3′NTR of the HCV genome is conserved through all genotypes^29^, we then generated the poly U/UC region of HCV genotype 2a JFH1 clone (Fig. 5e) and performed the RNA-binding assay under the same conditions. Biotin labeled poly U/UC RNA of HCV genotype 2a also bound to Hsc70 and the increasing amount of unlabeled poly U/UC RNA decreased the association to Hsc70 (Fig. 5e) as poly U/UC RNA from HCV genotype 1b (Fig. 5d).

### Tylophorine analogs stimulate the ATPase activity of Hsc70 in the presence of poly U/UC RNA

The association between Hsc70 and interacting proteins or RNAs is primarily regulated by its ATPase activity^19^. We determined the ability of tylophorine analogs to regulate the ATPase activity of Hsc70. DCB-3503 and *rac*-crytopleurine stimulated ATP hydrolysis of FL Hsc70 only at relatively high concentrations (Figure S4a and b). Meanwhile, the *K*_m_ of Hsc70 for ATP-binding was reduced to about 50% by DCB-3503 or *rac*-cryptopleurine in the presence or absence of poly U/UC RNA (Fig. 5f and g). Addition of an equal amount of poly C RNA into the reaction did not change the *K*_m_ for ATP-binding by DCB-3503 and *rac*-cryptopleurine under the same conditions (Fig. 5f and g). Hsp70 inhibitor 2-phenylethanesulfonamide (PES)^25^ did not change *K*_m_ (Fig. 5h), while PES inhibited hydrolysis of ATP by increasing *K*_m_ for ATP-binding in the presence of poly U/UC RNA (Fig. 5h). The *V*_max_ of Hsc70 for ATP hydrolysis did not change significantly under the same conditions by DCB-3503, or *rac*-cryptopleurine, or PES (Figure S4c–e).

### Co-chaperone protein BAG1 is involved in the anti-HCV activity of tylophorine analogs

**Figure 6:**
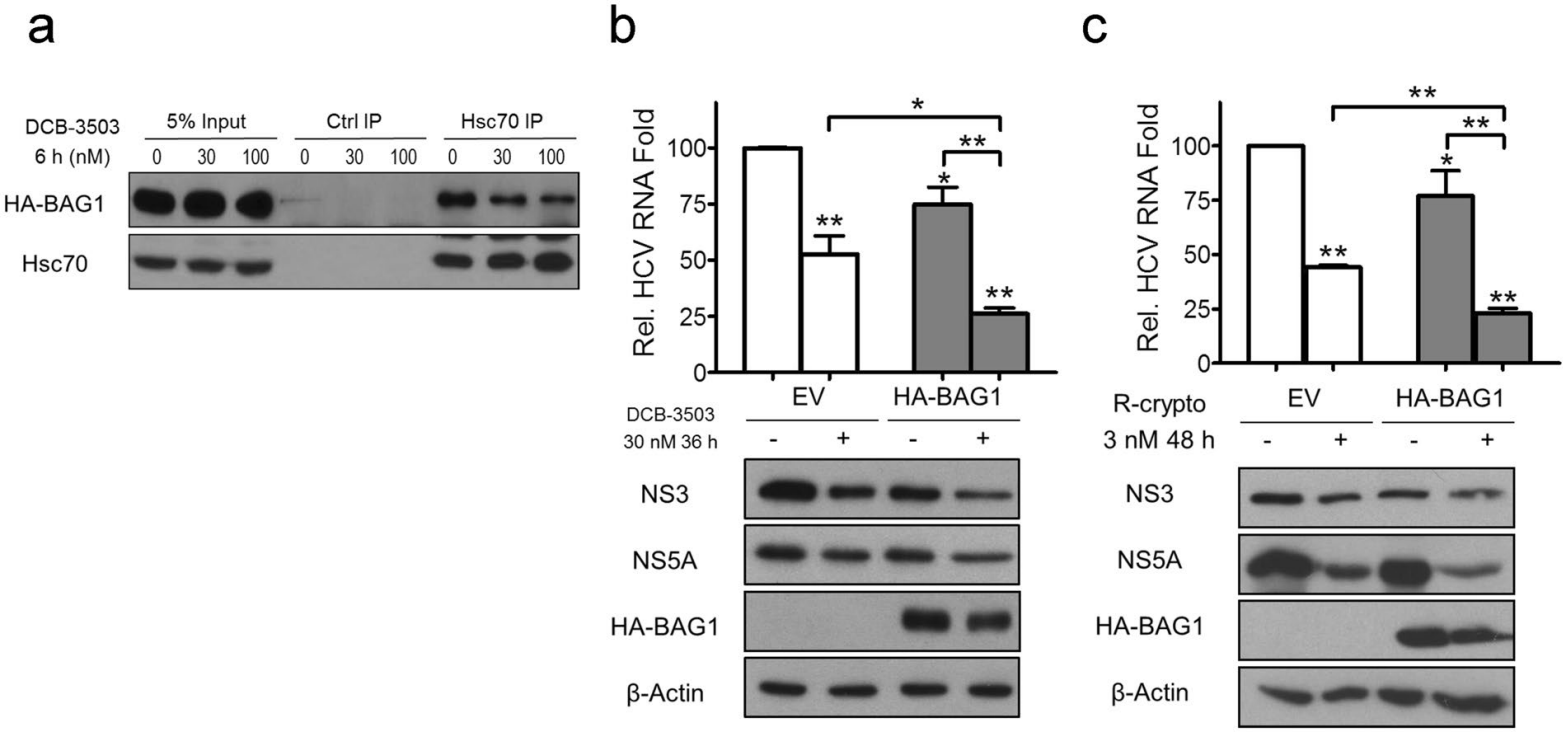
Co-chaperone protein BAG-1 regulate anti-HCV activity of tylophorine analogs. (**a**) DCB-3503 treatment decreased interaction of co-chaperone protein BAG-1 and Hsc70. Huh-luc/neo-ET cells were transiently transfected with HA- BAG-1 plasmid. The cells were treated with 30 nM DCB-3503 48 hours after transfection. Hsc70-associated HA-BAG-1 was immunoprecipitated with Hsc70 antibody as shown in Fig. 5a. Protein levels of HA-BAG-1 and Hsc70 were analyzed by Western blot. Huh-luc/neo-ET cells were transiently transfected in a six-well plate with 400 ng HA- BAG-1 plasmid or empty vector alone or in combination with (**b**) 30 nM DCB-3503, or (**c**) 3 nM *rac*-cryptopleurine for 36 hours. Levels of HCV RNA, NS3, NS5A, and HA-BAG-1 were analyzed by real-time PCR and Western blot, respectively. β-Actin served as the internal loading control. Results are representative of at least three independent experiments and presented as mean ± SD. *P* values are labeled on the graph.

Co-chaperone proteins regulate the ATPase activity of Hsc70 by modulating the ATP/ADP hydrolysis cycle^30^. The co-chaperone protein BAG-1 is bound to a conserved BAG domain of Hsc70 that interacts with its ATPase domain^30^. To determine the function of BAG1 in the anti-HCV activity of the tylophorine analogs, we constructed an HA-tagged BAG1 plasmid and transfected it into Huh-luc/neo-ET cells. The expression level of BAG1 was not affected by DCB-3503 treatment for up to 6 hours (Fig. 6a); however, the amount of BAG1 that bound to Hsc70 was decreased with the DCB-3503 treatment (Fig. 6a). Transfection of HA-tagged BAG1 enhanced the inhibitory activity of DCB-3503 and *rac*-cryptopleurine on HCV RNA replication, as well as expression levels of NS3 and NS5A (Fig. 6b,c). The expression level of HA-BAG1 was decreased with DCB-3503 and *rac*-cryptopleurine treatment for 48 hours (Fig. 6b,c).

## Discussion

The tylophorine analogs have exhibited various activities, yet the only molecular target VEGFR2 revealed for this group of compounds was associated with their anti-cancer activities^9^. One report has suggested that tylophorine or (–)-(*R*)-tylophorine attenuated HCV IRES-mediated translation by suppressing expression of cyclin A2^31^. However, results of our structure–activity relationship studies suggested that tylophorine was not a functional analog of DCB-3503 and *rac*-cryptopleurine^8, 21–23^. Our study revealed that DCB-3503 stimulated the ATPase activity of Hsc70, thus inhibited translation of cyclin D1 mRNA^10^. In this study, we evaluated both five- and six-membered E-ring tylophorine analogs as potent inhibitors of HCV replication (Figures 1 and S1a). No resistant clones could be isolated with either DCB-3503 or *rac*-cryptopleurine treatment (Figure S1a), suggesting that the tylophorine analogs inhibit HCV replication through modulation of the functions of the host’s factor(s).

### Hsc70 is associated with the HCV replication complex

Host chaperones and co-chaperones play important functions in the assembly of the viral replication complex and the replication of the viral genome. Hsc70 participated in the RNA-dependent replication of sindbis virus^32^, influenza virus^33^, rotavirus^34^, and dengue virus^35^. Hsc70 also associated with lipid droplet in HCV-infected cells^36^. Suppression of Hsc70 expression using shRNAs decreased the infectivity of the HCV J6/JFH virion^36^. DNAJC14 regulated the chaperone activity of Hsp70; over-expression of DNAJC14 disrupted the stoichiometry of the chaperone/substrate and assembly of the yellow fever virus replication complex^37^.

We demonstrated that HCV RNA mediated the binding of Hsc70 to the HCV replication complex (Figs 4 and 5). Part of Hsc70 exhibited the same sedimentation with NS3, NS5A, and HCV RNA in the continuous gradient fractions (Fig. 4a,b). The association of Hsc70 to the membrane-rich fraction containing the HCV replication complex was disrupted by changing the ionic strength or RNase treatment, which suggested that the association was dependent on RNA (Fig. 4d and S3c). DCB-3503 treatment perturbed the association of NS5A and NS5B to Hsc70 (Figures 5a and S3d,e), but increased the amount of HCV RNA bound to Hsc70 (Fig. 5a). Hsc70 specifically bound to the poly U/UC RNA motif in both HCV genotypes 1b and 2a (Figs 5b–e and S3f,g). The poly U/UC RNA motif is critical for the efficient translation and replication of HCV RNA efficiently^38, 39^. Deletion of the poly U/UC sequence inhibited replication of HCV in subgenomic replicon cells (Figure S3f)^29, 38^. An HCV viral genome lacking this element was not infectious^40^. Therefore, we presume that DCB-3503 and *rac*-cryptopleurine could also inhibit replication of other genotypes, for example HCV genotype 2a and its quasispecies that maintain the 3′NTR poly U/UC motif. Poly U/UC was essential for binding to replication factors like the FUSE binding protein^41^ and hnRNP A1^42^. These RNA-binding proteins regulated translation through either stabilization of RNA or promotion of RNA decay^43^. A single nucleotide base change sharply reduced the binding affinity between Hsc70 and its associated AUUUA motif in the 3′ untranslated region of host mRNAs^10^. We found that tylophorine analogs did not inhibit replication of HIV or HBV (results not shown). HIV and HBV have very different sequences from that of HCV, especially in that they do not contain the poly U/UC motif in the 3′NTR. These results suggest that Hsc70 is associated with HCV replication complex preferentially through binding to the uridine rich motif in the 3′NTR.

Two previous reports demonstrated that suppression of Hsc70 by using siRNAs or small molecule inhibitors did not affect HCV RNA replication but rather impacted on the release of virons^36, 44^. The conflict results may be due to the genotype difference of the Con1 isolate used in the present study from that of the J6/JFH isolated with infectivity^36, 44^. In addition, sequence and structure differences in the regulatory elements of HCV, like 5′NTR and 3′NTR may require different regulating elements that contribute to differential infectivity and replication^45^. Therefore, it is reasonable that we obtained results there were different from the previous reports^36, 44^.

### Hsc70 is involved in HCV replication through regulating the translation of HCV RNA

We identified Hsc70 as one of the molecular targets of functional tylophorine analogs using biotinylated-DCB-3503 and -*rac*-crytopleurine (Figures 2 and S1). The function of Hsc70 is regulated by communications between NBD and SBD. The ATP-bound form of Hsc70 has a “loose” configuration with low affinity for substrates; the ADP-bound state of Hsc70 shows high affinity with associated proteins or RNA^46^, and favors degradation of substrates^47^. For example, binding of Hsc70 but not Hsp70 to CHIP promoted its degradation through ubiquitination^48^. ATP hydrolysis of Hsc70 NBD induces a conformational switch in the adjacent SBD that enhances its binding affinity to substrates.

The binding between Hsc70 and tylophorine was affected by ATP concentration (Fig. 2b,c). DCB-3503 and *rac*-cryptopleurine bind to the D206S ATPase mutant form of Hsc70, suggesting that the functional ATPase pocket was not required for the interaction (Fig. 2e). Either attenuation of Hsc70 expression level or ectopic expression of the WT or D206S mutant form of Hsc70 enhanced the anti-HCV activity of DCB-3503 (Figs 3b–d and S2a,b, and d). The binding of DCB-3503 or *rac*-crytopleurine to Hsc70 stimulated its ATPase activity in the presence of the poly U/UC motif of HCV RNA (Fig. 5f,g) without affecting Hsc70 protein expression (Figure S1g). Over-expression of co-chaperone protein BAG-1, which regulates the chaperone activity of Hsc70 by promoting ATP hydrolysis function of Hsc70^49^, significantly enhanced the anti-HCV activities of DCB-3503 and *rac*-cryptopleurine (Fig. 6b,c). Therefore, modulating the homeostasis of the ATP/ADP hydrolysis cycle of Hsc70 by tylophorine analogs is related to the inhibitory activity to HCV RNA replication. The HCV RNA associated to Hsc70 in the ADP binding state may be sent to degradation as other substrates of Hsc70. Suppression of Hsp70 expression by its inhibitor, quercetin, attenuated the IRES-mediated translation of HCV J6/JFH virus^50^. It is also possible that perturbation of Hsc70 function markedly reduced the activities of NS5A and NS5B in the reduced level of free HCV RNA. The around 1 nM concentration of DCB-3503 accumulated in each fraction of fractions 5–12 (Fig. 4c) revealed that DCB-3503 could be associated with protein or a subcellular complex instead of existing in the free form. The concentration should be decreased sharply from fraction 3–12 if DCB-3503 was in the free form. Only co-localization of DCB-3503, NS proteins, and Hsc70 could make this possible. Regulating the ATPase activity of Hsc70 by DCB-3503 and *rac*-cryptopleurine may be one of the mechanisms by which these compounds modulate HCV replication.

### Allosteric regulation of Hsc70 is an alternative strategy to regulate HCV replication

ATPase activity of Hsc70 could be regulated by co-chaperones and/or small molecules, including the Bag (Bcl 2-associated athanogene) family of proteins^30, 49^, the Hsp40 family proteins^51^, the immunosuppressive agent 15-deoxyspergualin (DSG)^52^, or lycoricidine and analogs^53, 54^. DSG is associated with the EEVD domain in the C-terminal of Hsc70 and Hsp90^55^. N-substituted benzyl matrinic acid derivatives, ( + )-lycoricidine and analogs inhibited HCV replication through suppression of Hsc70 expression^53, 54, 56^.

Hsc70 is an abundant cellular protein; therefore, ectopic expression of Hsc70 could not suppress HCV replication (Fig. 3b–d)^57^. Constitutive deletion of Hsc70 is embryonically lethal in mice, causing defects in multiple organs^58^. We also observed that knockdown of Hsc70 by combined shRNAs induced cell death. Thus, allosterically modulating ATPase activity of Hsc70 could be a better option to perturb its function rather than decreasing its expression. DCB-3503 and *rac*-cryptopleurine binds to the NBD of Hsc70 but not HSP90 nor Hsp70 (Figures 2 and S1d) without changing the expression of Hsc70 (Figure S1g). Ectopic expression of BAG1, which promoted ATP hydrolysis activity of Hsc70^30^, significantly enhanced the anti-HCV activity of tylophorine analogs (Fig. 6). The allosteric regulation of Hsc70 by DCB-3503 and *rac*-cryptopleurine is distinct from that of existing Hsc70 inhibitors and has not been reported previously. To our knowledge, the tylophorine analogs are the first known class of small molecules that associated with the NBD of Hsc70 and promote the hydrolysis of ATP (Fig. 5f and g).

Taken together, we propose the following model for the mechanisms of action for the anti-HCV activity of functional tylophorine analogs. DCB-3503 or *rac*-cryptopleurine binds to the NBD of Hsc70 independent of the intact ATP pocket. The binding stimulates the ATP hydrolysis of Hsc70 in the presence of the poly U/UC motif of HCV RNA, and thus inhibits translation. The tylophorine analogs may be potentially used with other anti-HCV agents to attenuate HCV replication. We demonstrated that DCB-3503 exhibited anti-cancer and anti-arthritis activities^4, 5, 10^; therefore, this group of tylophorine analogs could have special advantages for the treatment of cancer and/or autoimmune diseases patients infected with HCV. However, many questions remain to be investigated. For example, the exact tylophorine binding site(s) of Hsc70 is (are) still unclear due to our failed attempt to determine the Hsc70-DCB-3503 or Hsc70-*rac*-cryptopleurine complex by crystallography. Will the specific binding to the poly U/UC motif in HCV genotype 2a also be related to anti-HCV activity? And, the dynamic regulation by the tylophorine analogs of ATP/ADP turnover in Hsc70 remains unexplained.

## Experimental procedures

### Materials and reagents

Cell culture media and fetal bovine serum (FBS) were purchased from Invitrogen. All reagents were purchased from Sigma-Aldrich (St. Louis, MO) except for those otherwise noted. The DCB series of compounds were synthesized in Dr. Baker’s laboratory. *rac*-Cryptopleurine and biotinylated-*rac*-cryptopleurine were synthesized in Dr. Alois Fürstner’s laboratory. The YXM series of compounds were synthesized in Dr. Kuo-Hsiung Lee’s laboratory. The PA series of compounds were provided by Dr. Shishan Yu^22^. 2-Phenylethanesulfonamide (PES) was purchased from Sigma–Aldrich. Information for plasmid construction and real-time PCR primers are provided in Tables S1 and S2, respectively.

### Affinity purification

The affinity purification method was adopted from a method reported by Emami *et al*.^59^. Cells were lysed in protein-binding buffer [PBB, 20Mm Hepes, pH 7.9, 100 mM NaCl, 0.5 mM EDTA, 0.5% Nonidet P-40, 6 mM MgCl_2_, 5 mM 2-mercaptoethanol, complete protease inhibitor (Roche)]. Biotinylated-*rac*-cryptopleurine and -DCB-3503 were bound overnight at room temperature to a 50% slurry of streptavidin–agarose beads (Invitrogen) in buffer containing 50% DMSO and 50% PBB. Beads were washed to remove unbound compound and then incubated with pre-cleared whole-cell lysates or recombinant protein in 2.5% BSA. Bound proteins were eluted, either specifically with *rac*-cryptopleurine or DCB-3503, or with a buffer specified in the figures. Proteins that remained bound to beads were eluted with SDS loading buffer. Samples were separated with SDS PAGE, and examined by Western blot or silver staining. Specific bands from silver-stained gel were analyzed by LTQ Orbitrap mass spectrophotometry (Yale University W.M. Keck Foundation Biotechnology Resource Laboratory).

### Statistical analysis

Data were analyzed by a two-tailed student’s *t*-test (Microsoft Office Excel). The difference was considered to be statistically significant when *p* < 0.05.

### Data availability

All data generated or analyzed during this study are included in this published article and its Supplementary Information files.

## Electronic supplementary material

Supplemental Information
