# Supplementary material for: Tylophorine Analogs Allosterically Regulates Heat Shock Cognate Protein 70 And Inhibits Hepatitis C Virus Replication
Source: Sci Rep. 2017 Aug 30;7:10037. doi: 10.1038/s41598-017-08815-z (PMC5577180; doi:10.1038/s41598-017-08815-z)
Supplement: Supplementary file 1 — Supplemental Information [file 41598_2017_8815_MOESM1_ESM.pdf]

Tylophorine Analogs Allosterically Regulates Heat Shock Cognate Protein 70 and Inhibits Hepatitis C Virus Replication

Ying Wang, Sangwon Lee, Ya Ha, Wing Lam, Shao-Ru Chen, Ginger E. Dutschman, Elizabeth A. Gullen, Susan P. Grill, Yao Cheng, Alois Fürstner, Samson Francis, David C. Baker, Xiaoming Yang, Kuo-Hsiung Lee, Yung-Chi Cheng

Supplemental Experimental Procedures

Cell lines and growth conditions

HCV genotype 1b Con1 subgenomic replicon cell lines Huh-luc/neo-ET containing a luciferase reporter, Huh 9-13, and HCV genotype 1b plasmid pFK-*I389*/NS3-3 were provided by Dr. Ralf Bartenschlager from The University of Heidelberg [1,2]. HCV genotype 2a genomic RNA JFH1 clone was a gift of Dr. Takaji Wakita (Tokyo Metropolitan Institute of Neuroscience, Tokyo Metropolitan Organization for Medical Research, Japan). Huh luc/neo-ET and Huh 9-13 cells were maintained in DMEM medium supplied with 10% FBS, 1 mM nonessential amino acids (Invitrogen), and 250 µg/ml G418 (Gibco). Generation of HLN-cure cells is described in supplementary material. HLNC cells were maintained in DMEM medium supplied with 10% FBS.

shRNAs and plasmids

The shRNAs for Hsc70 were designed by the BLOCK-iT™ RNAi Designer provided by Invitrogen and cloned into BLOCK-iT™ inducible H1 RNAi entry vector (Invitrogen). The complementary DNA oligonucleotides as follows: Hsc70 shRNA-1 (5’-

GCTGTTGTCCAGTCTGATATGcgaaCATATCAGACTGGACAACAGC-3’) (SENSE-loop-ANTISENSE), Hsc70 shRNA-2 (5’-GCCCAAGGTCCAAGTAGAATAcgaaTATTCTACTTGGACCTTGGGC-3’) (SENSE-loop-ANTISENSE).

The HCV pFKI389 lucubineo NS3-3' plasmid was a generous gift from Dr. Ralf Bartenschlager at the University of Heidelberg [1].

The Hsc70 NBD (residues 1-385) plasmid was constructed by inserting a stop codon after position 1155 in the Hsc70 FL pET28a plasmid using the QuikChange® XL site-directed mutagenesis kit (Stratagene). The D206S mutation in the Hsc70 gene, rHsc70-1, rHsc70-2, and the D318N mutation in the NS5B gene were introduced using the QuikChange® XL site-directed mutagenesis kit. The ER-Hsc70 plasmid was constructed by inserting the ER localization signal peptide (MSFVSLLLVGILFWATEQLTKCEVFQ) into the N-terminus of HA-Hsc70 plasmid. Primers used for mutagenesis are shown in Table S1. Poly A was cloned from synthetic poly A into a pGL4 vector. To generate the HCV pFKI389 lucubineo NS3-3' plasmid without the poly U/UC motif at the 3’ NTR, we cloned the plasmid without the poly U/UC motif by adding a Sca I site to the end of the NS5B coding region, and ligated the linear DNA by the new Sca I site and the existing Sca I site after the poly U/UC motif.

Generation of HLN-cure cells

Highly permissive HLN-cure cells were derived by through interferon treatment similar to the derivation of Huh 7.5 cells described previously [3]. Briefly, Huh-luc/neo-ET cells were passaged twice in the absence of G418 and then cocultured with 100 IU/ml of recombinant human IFNα-2a (Pestka Biomedical Laboratories) for 3 days. On the fourth day, cells were passaged and cultured for 24 hours before re-introduction of IFNα-2a. Cells were then passaged for eight times in the presence of IFNα-2a and rested at every

fourth passage. The elimination of HCV RNA was monitored by Real-Time PCR. HLN-cure cells, verified to be free of HCV RNA, were propagated and expanded without IFNα-2a. Electroporation was conducted using HLN-cure cells that were passaged fewer than 20 times from the cryopreserved HLN-cure stocks.

Transfection of plasmids

All plasmids were cleared of endotoxins using the EndoGO kit (MirusBio, Madison, WI) prior to transfection. Transfection was performed with TransIT-LT1 transfection reagent (MirusBio) for Huh-luc/neo-ET cells according to the manufacturers' instructions. HCV mRNA was transfected into Huh 7.5 cells by electroporation according to the protocol described previously [4].

Expression and purification of recombinant Hsp70, full length and different domains of Hsc70

Primers for the construction of expression vectors of for His-tagged Hsp70, full-length Hsc70, and different Hsc70 domains are shown in Table S1. Recombinant proteins were overproduced in *E.coli* BL21 (DE3) pLysS cells (Promega, Madison, WI) at 22°C for 4 hours, and purified by Ni^{2+}-NTA agarose (Qiagen, Valencia, CA).

Determination of anti-HCV activity by luciferase reporter assay

Firefly-luciferase reporter activity was used to monitor the replication of HCV replicons in Huh-luc/neo-ET cells free from G418 [5]. Luciferase activity was measured with a luciferase assay kit (Promega) on Tecan FARCyte Luminometer (GE Healthcare) according to the manufacturer's instruction.

RNA isolation and Real-Time PCR

Total RNA was isolated using the High Pure RNA isolation kit (Roche, Mannheim, Germany). cDNA was synthesized from total RNA using random primers according to the protocol of the iScript^{TM} cDNA synthesis kit (Bio-Rad Laboratories, Hercules, CA), and expression levels of specific genes were quantified by Real-Time PCR with gene-specific primers (Table S5) using the SsoFast^{TM} EvaGreen supermix (Bio-Rad Laboratories). Absolute copy number of HCV RNA was determined with the standard curve generated using the linearized pFK-*I389*/NS3-3′ plasmid bearing the HCV genome and 18S rRNA was included as a control.

***In vitro* transcription assay**

HCV subgenomic replicon encoding plasmid pFK I389 lucubineo NS 3-3' was linearized by ScaI. Different fragments of the 3' NTR region from HCV RNA were cloned into a TA-PCRII vector (Invitrogen) with a T7 promoter. Primers used for cloning are provided in Table S1. The linearized plasmids were used as the template for *in vitro* transcription. Uncapped and capped HCV replicon mRNAs were generated using the MEGAscript kit containing T7 RNA polymerase (Ambion, Austin, TX). The *in vitro* transcribed mRNAs were purified using the MEGAclear kit (Ambion). The integrity of the *in vitro* transcribed mRNAs was confirmed using the Agilent 2100 Bioanalyzer (Foster City, CA). The purified mRNAs were used for *in vitro* translation experiments, or *in vitro* RNA binding assays, or ATPase hydrolysis assays.

Antibodies

The following antibodies were used: rabbit and mouse IgG, anti-Alix (2171, Cell Signaling), anti-β-Actin (A5316, Sigma-Aldrich), anti-Calnexin (2679, Cell Signaling), anti-GRP78 (G8918, Sigma-Aldrich), anti-Hrs mAb (ab56468, Abcam), anti-Hsc70 (sc-7298, Santa Cruz; SPA-815, Stressgen), anti-Hsp70 (ab2787, Abcam), anti-Hsp90

(SPA-830, Stressgen), anti-NS3 (ab65407, Abcam; MAB8691, Millipore), anti-NS5A (sc-52417, Santa Cruz; MAB8694, Millipore), anti-NS5B (ab100895, Abcam), anti-HA (sc-7392, Santa Cruz), and biotin conjugated Rat-IgG1 (200005-11-B, Alpha Diagnostic International). All antibodies were used at a 1:2,000 dilution for Western blot analysis.

Optiprep iodixanol continuous density gradient

Optiprep iodixanol density media was purchased from Sigma-Aldrich. Continuous density gradients were performed as described [6]. Briefly, 10^7 Huh-luc/neo-ET cells were washed 3 times in ice cold PBS and resuspended in 1 ml hypotonic buffer containing 0.25 M sucrose, 4 mM $MgCl_2$, 8.4 mM $CaCl_2$, 10 mM EDTA, 50 mM Hepes-NaOH at pH 7.0 with complete protease inhibitor, 4% ribonucleoside vanadyl, and 400 U RNase Out (Invitrogen). Cells were then lysed by 30 passages through a 20-gauge needle, and centrifuged three times at 1,000 g for 5 minutes to remove cellular debris and nuclei. Continuous 5-30% optiprep iodixanol gradients were prepared in 78 mM KCl, 4 mM $MgCl_2$, 8.4 mM $CaCl_2$, 10 mM EDTA, and 50 mM Hepes-NaOH at pH 7.0 using a gradient mixer and stored at 4 °C. Post-nuclear supernatant was loaded on top of the gradient media, and centrifuged at 36,000 rpm at 4°C using a SW 41Ti Beckman Coulter rotor for 80 minutes. Each sample was collected and divided into 12 fractions by an Autodensi-Flow Gradient Fractionator.

Preparation of HCV crude replication complexes (CRCs)

Preparation of CRCs was adapted from a previous protocol with modification [7]. All reagents were RNase-free. Ten million Huh 9-13 or Huh luc/neo-ET cells were incubated with 1 ml ice-cold hypotonic buffer containing 0.25 M sucrose, 4 mM $MgCl_2$, 8.4 mM $CaCl_2$, 10 mM EDTA, 50 mM Hepes-NaOH at pH 7.0 with complete protease inhibitor, 4% ribonucleoside vanadyl, and 400 U RNase Out. After sitting on ice for 30 minutes,

cells were lysed by 30 passages through a 20-gauge needle, and centrifuged three times at 1,000 g for 5 minutes at 4°C to remove cellular debris and nuclei. The supernatant was centrifuged in a Beckman TL-100 ulatracentrifuge with TLS-55 rotor at 15,000 g for 20 minutes at 4 °C, and the resulting pellet was used as CRCs.

Immunoprecipitation of Hsc70-associated protein and mRNA complexes (RNA-IP)

RNA-IP was performed as described by Keene *et al* [8]. Briefly, CRC from Huh 9-13 or Huh-luc/neo-ET cells was lysed in polysome lysis buffer [100 mM KCl, 5 mM $MgCl_2$, 10 mM Hepes, pH 7.0, 0.5% NP 40, 1 mM DTT, 100 U/ml RNase inhibitor, 2 mM ribonucleoside vanadyl (New England Biolabs), complete protease inhibitor]. Supernatant was immunoprecipitated with biotin-conjugated anti-Hsc70 antibody and Dynabeads® MyOne^{TM} Streptavidin T1 beads (Invitrogen) at 4°C overnight. Biotin-conjugated Rat-IgG1 was used as an antibody isotype control. Beads were washed with NT 2 buffer (50 mM Tris-HCl, pH 7.4, 150 mM NaCl, 1 mM $MgCl_2$, 0.05% NP 40) for four times before the addition of SDS loading buffer for protein analysis or treatment with 30 μg proteinase K in NT 2 buffer at 50°C for 1 hour before RNA isolation. Bound RNA was isolated by phenol-chloroform extraction, and glycogen-sodium acetic-ethanol precipitation. Bound protein was examined by Western blot. Hsc70-bound RNA was analyzed by real-time PCR.

***In vitro* RNA binding assay**

For the *in vitro* RNA binding assay, 100 μM *in vitro* transcribed biotinylated HCV RNA (RNA sequence was shown in Figure 5B) was incubated with 150 μM Hsc70 in RNA binding buffer (10 mM Tris HCl pH 7.4, 50 mM KCl, 1.5 mM $MgCl_2$, 25 mM NaCl, 0.5 mM DTT, 0.5 μg/ml yeat tRNA, 0.05% NP-40, and 2.5% BSA) at 4 °C for 30 minutes. The

RNA-protein complex was eluted by addition of 20 µl streptavidin beads and resolved by SDS PAGE and Western blot analysis. For the competition assay, increasing concentration of *in vitro* transcribed unlabeled RNA was added into the assay along with the biotinylated RNA.

Liquid chromatography-mass spectrometry (LC-MS)

LC-MS/MS was performed according to the protocol published before [9]. Proteins in each fraction obtained from the iodixanol gradient centrifugation were removed by acetonitrile: methanol (2:1) precipitation. The aqueous phase was dried, and reconstituted to 100 µl using acetonitrile:water solution (12:88). The chromatographic separation was achieved on a ZORBAX 3.5 µM extended-C_{18} column (2.1 × 50 mm, Agilent, Palo Alto, CA) on the Agilent 1200 HPLC series (Agilent). The ionized mass was detected using the Applied Biosystems Sciex 4000 Q-trap® mass spectrometer (Applied Biosystems). Data acquisition was carried out by Analyst 1.4.2® software. Quantification was performed using multiple reactions monitoring (MRM) of the transitions of m/z 410 to m/z 392 for DCB-3503, and m/z 378 to m/z 84 for *rac*-cryptopleurine.

Isothermal titration calorimetry (ITC) assay

ITC measurements were carried out on a VP-ITC microcalorimeter (GE healthcare MicroCal) in the Biophysics Resource of the W.M. Keck Biotechnology Research Laboratory at Yale University. Nucleotide-free protein solutions were dialyzed overnight against a buffer containing 10mM sodium Hepes and 50 mM NaCl (pH 7.5) at 4°C. The compounds were dissolved in 100% DMSO and their concentrations compounds were calculated based on the reported extinction coefficient of tylophorine at 257nm. All the measurements were done in dialysis buffer containing 5% DMSO at 25°C. The ITC experiment was done with 15 µM protein in the cell and 170 µM DCB-3503 or *rac*-

cryptopleurine in the syringe. There were 20 injections in total; the first injection was 3 µL and the remaining 19 were 15 µL each, with 240 seconds spacing in between injections. The thermogram was analyzed using the Microcal ORIGIN 7.0 software supplied with the instrument.

Characterization of ATP hydrolysis activity of Hsc70

Characterization of the ATP hydrolysis activity of Hsc70 was described previously [10]. Briefly, 0.25 µM Hsc70 was incubated in the presence or absence of various concentrations of compound, ATP, and 0.5 µM RNA in 50 µl reaction buffer [25 mM MOPS, pH 7.4, 10 mM $Mg(OAc)_2$, 30 mM KOAc, 2 mM DTT, 1 mg/ml BSA] at 37°C for 10 minutes. Short poly U/UC RNA and its complementary RNA strand poly C RNA were *in vitro* transcribed and purified. The reaction was stopped by adding 45% trichloroacetic acid to a final concentration of 15% (v/v). The aqueous phase was washed with 1,1,2-trichlorotrifluoroethane: trioctylamine (55:45, v/v) twice. The concentrations of ATP and ADP in the aqueous phase were analyzed by HPLC.

Supplemental Information Legend

Figure S1 (related to Figures 1 and 2). Tylophorine analogs interacted with Hsc70. (A) Huh-luc/neo-ET cells were incubated with 300 nM DCB-3503, 30 nM *rac*-cryptopleurine, and 200 nM *meso*-tetrakis-porphyrin compound 6 for three weeks. Clones were stained by 0.5% methylene blue in 50% ethanol. (B) Chemical structures of DCB-3503, *rac*-cryptopleurine, and their biotinylated analogs. (C) Workflow of affinity purification using biotinylated-*rac*-cryptopleurine or -DCB-3503. (D) Hsc70 was eluted by 0.1% and 0.5% Nonidet P-40, 0.4 M NaCl. (E) Recombinant his-tagged HSC70 and HSP70 was probed with HSC70 antibody and his tag antibody. (F) ITC results of the binding between DCB-3503 or *rac*-cryptopleurine to FL and NBD of Hsc70. (G) Hsc70 protein level under the treatment of DCB-3503 and *rac*-cryptopleurine in HCV replicon cells. β-Actin serves as an internal control for protein loading. All results are representative of at least three independent experiments.

Figure S2 (related to Figures 3 and 4). Expression levels of proteins in Figure 3B - 3D were quantitated in (A), (B), and (D), respectively. Growth curve of Huh-luc/neo-ET cells transfected with (C) Hsc70 shRNAs and (E) WT HA and D206S mutant Hsc70 plasmids. (F) Treatment of DCB-3503 and *rac*-Cryptopleurine inhibited translation of HCV pFKI 389 lucubineo NS 3-3’ RNA in rabbit reticulocyte lysate *in vitro* translation system. (G)Transient replication assay of pFK I389 NS3-3’ HCV RNA into HLNcure cells pre-treated with 30 nM DCB-3503 24 hours prior to electroporation. HCV replication after electroporation was monitored by luciferase activity. (H) Expression level of Hsc70 was examined using Western blot analysis before and after electroporation. (H) The density of the fractions obtained using optiprep iodixanol density gradient centrifugation. All results are representative of at least three independent experiments. Results in (A) - (G) are presented as mean ± S.D. (* $p < 0.05$, ** $p < 0.01$)

Figure S3 (related to Figure 5). Analysis of Hsc70 bound HCV NS proteins. (A) Huh 9-13 cells were transiently transfected with HA-tagged NS3/4A or NS4B plasmids. Cells were harvested and lysed 48 hours after transfection. The associations of HA-NS3/4A and HA-NS4B with Hsc70 were examined by immunoprecipitation as described in Figure 4A. (B) Hsc70-associated complex was immunoprecipitated with Hsc70 antibody. The levels of surivin and β-actin RNA were examined by real-time PCR as in Figure 5B. (C) The Hsc70-associated complex was treated with 100 μg/ml RNase for 2 hours before subject to SDS PAGE and Western blot analysis of NS5A and NS5B levels. (D) Huh 9-13 cells were transiently transfected with HA-tagged NS5B plasmids. Cells were harvested and lysed 48 hours after transfection. The associations of HA-NS5B with Hsc70 were examined by immunoprecipitation as described in Figure 4A. (E) Huh 9-13 cells were treated with DCB-3503 for the time and dose indicated on the figure before harvest, biotinylated-HCV RNA was used to pull down associated proteins. The presence of NS5B and Hsc70 in the HCV RNA-associated complex was determined by western blot analysis. (F) Streptavidin beads alone or *in vitro* transcribed biotinylated short poly U/UC RNA was mixed with different fragments of recombinant Hsc70 (full length 1-647, NBD 1-385, and SBD 376-647). RNA-bound protein was detected using Western blot analysis. (G) Streptavidin beads alone or *in vitro* transcribed biotinylated short poly U/UC RNA was mixed with purified recombinant Hsc70 or Hsp70 protein. RNA-bound protein was detected by Western blot analysis. (H) The numbers of G418-resistant clones were scored three weeks after electroporation from wild type and poly U/UC deleted (Δ U/UC) pFK-I389/NS3-3'/LucUbiNeo-ET constructs. Each number is the average obtained from three electroporation experiments. The bar shows the standard deviation.

Figure S4 (related to Figure 5). (A) DCB-3503 and (B) *rac*-Cryptopleurine stimulated the ATPase activity of FL Hsc70. Effect of (C) DCB-3503, (D) *rac*-cryptopleurine, or (E)

2-phenylethanesulfonamide (PES) on V_{max} of Hsc70 for ATP in the presence or absence of short poly U/UC or poly C RNA. The concentration of the ADP generated was analyzed and calculated based on the area under the curve (AUC) with a standard curve. The K_m and V_{max} values were calculated by fitting the data into Michaelis-Menten kinetics equation. Error bars represent standard deviations from at least three independent experiments.

Table S1. Primers and vectors used for plasmid construction.

	Primer (Forward/ Reverse, 5'-3')	Restriction sites	Vector
HA-Hsc 70	AAATATATCTCGAGGATGTCCAAGGGACCTGCAGTTGGTA/<br>AAATATATGGATCCTTAATCAACCTCTTCAATGGTGGGC	XhoI<br>BamHI	pcDNA5/TO
His-Hsc 70	AAATATATCATATGATGTCCAAGGGACCTGCAGTTGGTA/<br>AAATATATCTCGAGATCATTAATCAACCTCTTCAATGGTGGGC	NdeI<br>XhoI	pET28a
His-Hsc70<br>(1-375)	GTCTGGAGACAAGTCTTAAGAGAATGTTCAAGATT/<br>AATCTTGAACATTCTCTTAAGACTTGTCTCCAGAC (mutagenesis)		
His-Hsc70<br>(376-647)	AAATATATGGATCCATGCAGGCAGCCATCTTGTCTGG/<br>AAATATATCTCGAGATTAATCAACCTCTTCAATGGTGGGC	BamHI<br>XhoI	pET28a
His-Hsc70<br>(376-550)	AAATATATGGATCCATGCAGGCAGCCATCTTGTCTGG/<br>AAATATATCTCGAGTTATTTCATGTTGAAGGCATAGG	BamHI<br>XhoI	pET28a
HA-D206S<br>Hsc70	GGAGGTGGCACTTTTAGTGTGTCAATCCTCACT/<br>AGTGAGGATTGACACACTAAAAGTGCCACCTCC (mutagenesis)		
His-Hsp70	AAATATATCATATGATGGCCAAAGCCGCGGCGAT/	Nde1	pET28a

	AAATATATCTCGAGACTAATCTACCTCCTCAATGG	Xho1	
HA-NS3/4A	AAATATATGGATCCGATGTACCCATACGATGTTCCAGATTACGCT ATGGCGCCTATTACGGCCTAC/ AAATATATTCTAGATCACTAGCACTCTTCCATCTCATCGAA	BamHI XbaI	pcDNA5/TO
HA-NS4B	AAATATATCTTAAGATGTACCCATACGATGTTCCAGATTACGCTGCCTCACACCTC CCTTACAT/ AAATATATTCTAGACTAGCATGGCGTGGAGCAGT	AflII XbaI	pcDNA5/TO
HA-NS5A	AAATATATCTTAAGATGTACCCATACGATGTTCCAGATTACGCTTCCGGCTCGTG G/ AAATATATTCTAGATCACTAGCAGCAGACGACGTCCTCACTAGC	AflII XbaI	pcDNA5/TO
HA-NS5B	AAATATATCTTAAGATGTACCCATACGATGTTCCAGATTACGCTTCGATGTCCTAC ACATGGAC/ AAATATATTCTAGATCATCGGTTGGGGAGTAGAT	AflII XbaI	pcDNA5/TO
1b AU+U/UC	AAATATATCTCGAGGTTGGGGTACCGCCCTTGCGA/ AAATATATGGATCCAGTCAAGCGGCTCACGGACC	XhoI BamHI	TA PCR II

1b AU	AAATATATCTCGAGGGCTGCGTGGGAGACAGCTAGA/	XhoI	TA PCR II
	AAATATATGGATCCTCATCGGTTGGGGAGTAGAT	BamHI	
1b Long	AAATATATCTCGAGACGGGGAGCTAAACACTCCA/	XhoI	TA PCR II
U/UC	AAATATATGGATCCAGTCAAGCGGCTCACGGACC	BamHI	
1b Short	AAATATATCTCGAGCTAAACACTCCAGGCCAATAGGC/	XhoI	TA PCR II
U/UC	AAATATATGGATCCGAGCCACCAAAGGAAAGAAAAGG	BamHI	
1b Poly A	AAATATATCTCGAGAAACCACAACTAGAATGCAGTGAA/	XhoI	TA PCR II
	AAATATATGGATCCGTTAACTTGTTTATTGCAGCTTAT	BamHI	
2a poly	AAATATATAGCGGCACACACTAGGTACA	XhoI	TA PCR II
U/UC	AAATATATACATGATCTGCAGAGAGACC	BamHI	
BAG1	AAATATATGGATCCGATGAACAGTCCACAGGAAGAGGT	BamHI	pcDNA5/TO
	AAATATATTCTAGATCACTCAGTCTCCTGGCAGATGT	XbaI	

Table S2. Primers for Real-Time PCR.

	**Forward Primer (5'-3')**	**Reverse Primer (5'-3')**
HCV	CGGGAGAGCCATAGTGGTCTGCG	CTCGCAAGCACCCTATCAGGCAGTA
Survivin	AGGACCACCGCATCTCTACAT	AAGTCTGGCTCGTTCTCAGTG
β-Actin	ATTGCCGACAGGATGCAGAA	GCTGATCCACATCTGCTGGAA

Table S3. EC_{50} of DCB-3503, *rac*-cryptopleurin, and their biotinlylated derivatives against HCV replication, and IC_{50} against Huh-luc/neo-ET cell.

	EC_{50} (nM)	IC_{50} (nM)
DCB-3503	31.5 ± 7.0	91 ± 11
Biotinylated-DCB-3503	> 500 µM	> 500 µM
rac-cryptopleurine	0.6 ± 0.1	2.0 ± 0.1
Biotinylated-*rac*-cryptopleurine	33.0 ± 2.0	105.1 ± 5.2

References

1 Vrolijk, J. M. *et al.* A replicon-based bioassay for the measurement of interferons in patients with chronic hepatitis C. *J Virol Methods* **110,** 201-209, (2003).

2 Lohmann, V. *et al.* Replication of subgenomic hepatitis C virus RNAs in a hepatoma cell line. *Science* **285,** 110-113, (1999).

3 Blight, K. J., McKeating, J. A. & Rice, C. M. Highly permissive cell lines for subgenomic and genomic hepatitis C virus RNA replication. *J Virol* **76,** 13001-13014, (2002).

4 Quinkert, D., Bartenschlager, R. & Lohmann, V. Quantitative analysis of the hepatitis C virus replication complex. *J Virol* **79,** 13594-13605, (2005).

5 Cheng, Y. *et al.* A novel class of meso-tetrakis-porphyrin derivatives exhibits potent activities against hepatitis C virus genotype 1b replicons in vitro. *Antimicrob Agents Chemother* **54,** 197-206, (2010).

6 Gibbings, D. J., Ciaudo, C., Erhardt, M. & Voinnet, O. Multivesicular bodies associate with components of miRNA effector complexes and modulate miRNA activity. *Nat Cell Biol* **11,** 1143-1149, (2009).

7 Yang, W., Sun, Y., Phadke, A., Deshpande, M. & Huang, M. Hepatitis C virus (HCV) NS5B nonnucleoside inhibitors specifically block single-stranded viral RNA synthesis catalyzed by HCV replication complexes in vitro. *Antimicrob Agents Chemother* **51,** 338-342, (2007).

8 Keene, J. D., Komisarow, J. M. & Friedersdorf, M. B. RIP-Chip: the isolation and identification of mRNAs, microRNAs and protein components of ribonucleoprotein complexes from cell extracts. *Nat Protoc* **1,** 302-307, (2006).

9 Zhang, W., Dutschman, G. E., Li, X., Ye, M. & Cheng, Y. C. Quantitation of Irinotecan and its two major metabolites using a liquid chromatography-electrospray ionization tandem mass spectrometric. *J Chromatogr B Analyt Technol Biomed Life Sci* **877,** 3038-3044, (2009).

10 Yamagishi, N., Ishihara, K. & Hatayama, T. Hsp105alpha suppresses Hsc70 chaperone activity by inhibiting Hsc70 ATPase activity. *J Biol Chem* **279,** 41727-41733, (2004).

Figure S1

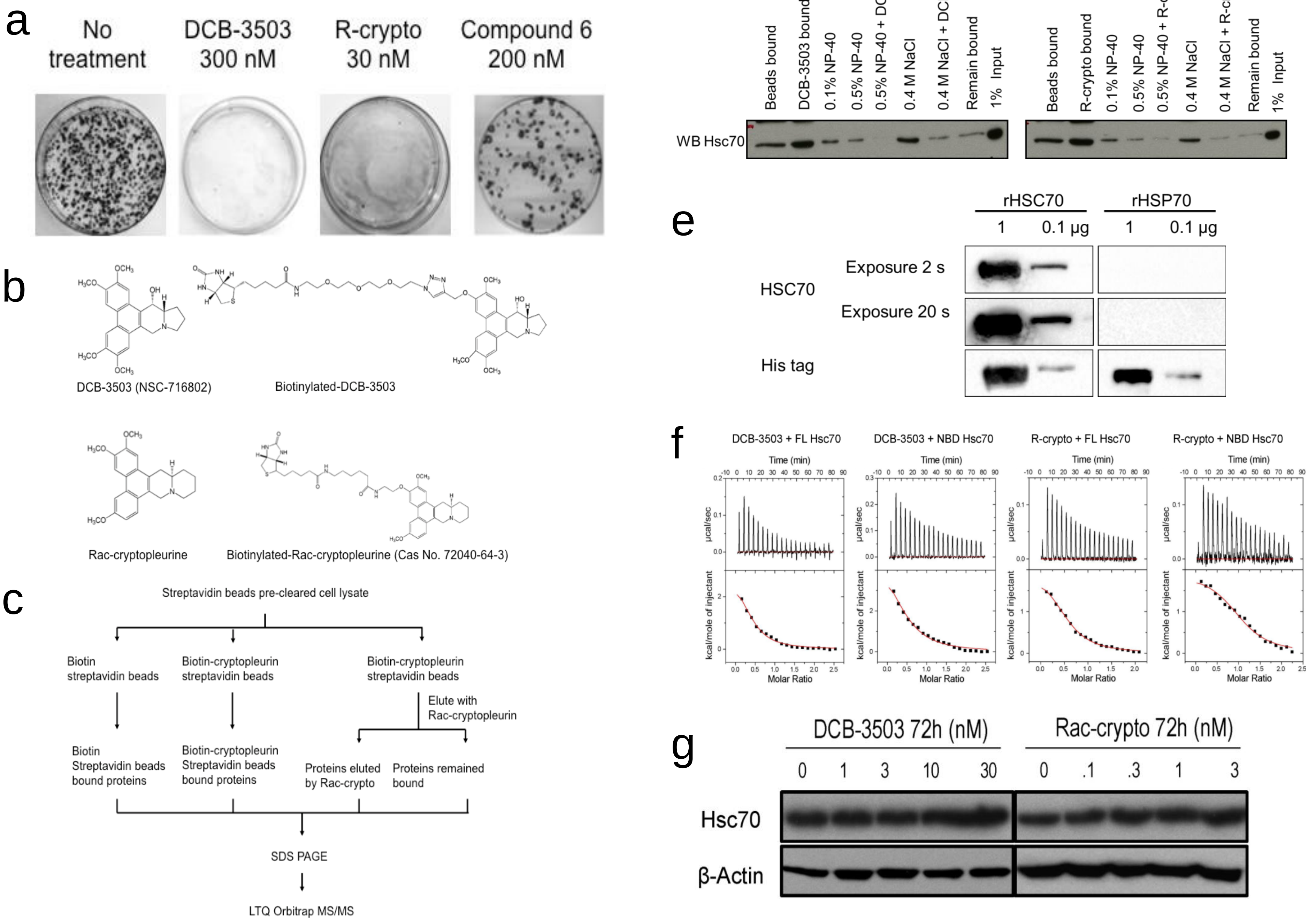

a
No treatment
DCB-3503 300 nM
R-crypto 30 nM
Compound 6 200 nM
b
DCB-3503 (NSC-716802)
Biotinylated-DCB-3503
Rac-cryptopleurine
Biotinylated-Rac-cryptopleurine (Cas No. 72040-64-3)
c
Streptavidin beads pre-cleared cell lysate
Biotin streptavidin beads
Biotin-cryptopleurin streptavidin beads
Biotin-cryptopleurin streptavidin beads
Elute with Rac-cryptopleurin
Biotin Streptavidin beads bound proteins
Biotin-cryptopleurin Streptavidin beads bound proteins
Proteins eluted by Rac-crypto
Proteins remained bound
SDS PAGE
LTQ Orbitrap MS/MS
d
Beads bound
DCB-3503 bound
0.1% NP-40
0.5% NP-40
0.5% NP-40 + DCB-3503
0.4 M NaCl
0.4 M NaCl + DCB-3503
Remain bound
1% Input
Beads bound
R-crypto bound
0.1% NP-40
0.5% NP-40
0.5% NP-40 + R-crypto
0.4 M NaCl
0.4 M NaCl + R-crypto
Remain bound
1% Input
WB Hsc70
e
rHSC70
rHSP70
1
0.1 µg
1
0.1 µg
HSC70
Exposure 2 s
Exposure 20 s
His tag
f
DCB-3503 + FL Hsc70
DCB-3503 + NBD Hsc70
R-crypto + FL Hsc70
R-crypto + NBD Hsc70
Time (min)
µcal/sec
kcal/mole of injectant
Molar Ratio
g
DCB-3503 72h (nM)
0 1 3 10 30
Rac-crypto 72h (nM)
0 .1 .3 1 3
Hsc70
β-Actin

Figure S2

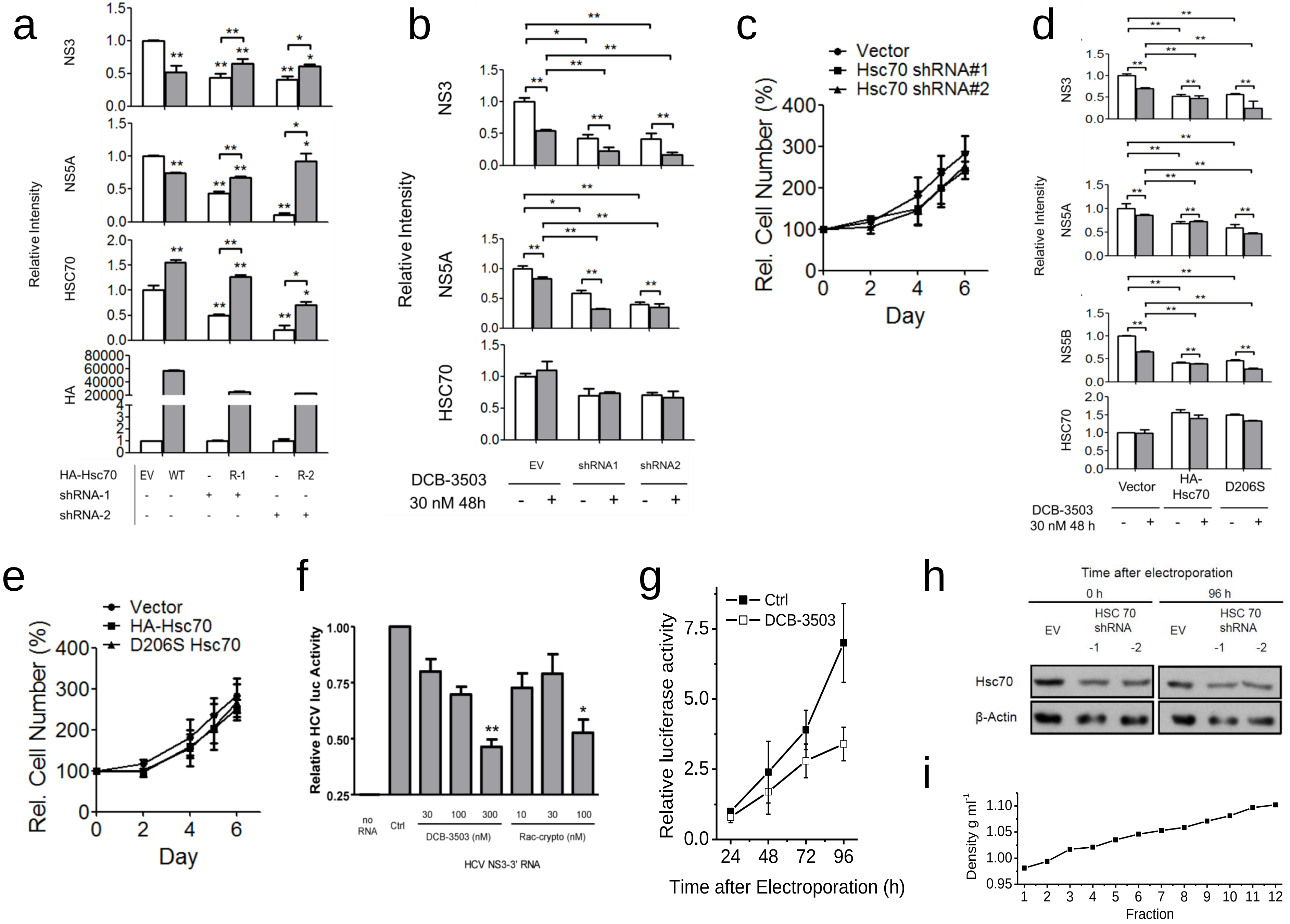
a
NS3
NS5A
HSC70
HA
Relative Intensity
HA-Hsc70 EV WT - R-1 - R-2
shRNA-1 - - + + - -
shRNA-2 - - - - + +
b
NS3
NS5A
HSC70
Relative Intensity
EV shRNA1 shRNA2
DCB-3503
30 nM 48h
c
Vector
Hsc70 shRNA#1
Hsc70 shRNA#2
Rel. Cell Number (%)
Day
d
NS3
NS5A
NS5B
HSC70
Relative Intensity
Vector
HA-Hsc70
D206S
DCB-3503
30 nM 48 h
e
Vector
HA-Hsc70
D206S Hsc70
Rel. Cell Number (%)
Day
f
Relative HCV luc Activity
no RNA
Ctrl
DCB-3503 (nM)
Rac-crypto (nM)
HCV NS3-3' RNA
g
Ctrl
DCB-3503
Relative luciferase activity
Time after Electroporation (h)
h
Time after electroporation
0 h
96 h
EV
HSC 70 shRNA
-1 -2
Hsc70
β-Actin
i
Density g ml-1
Fraction

Figure S3

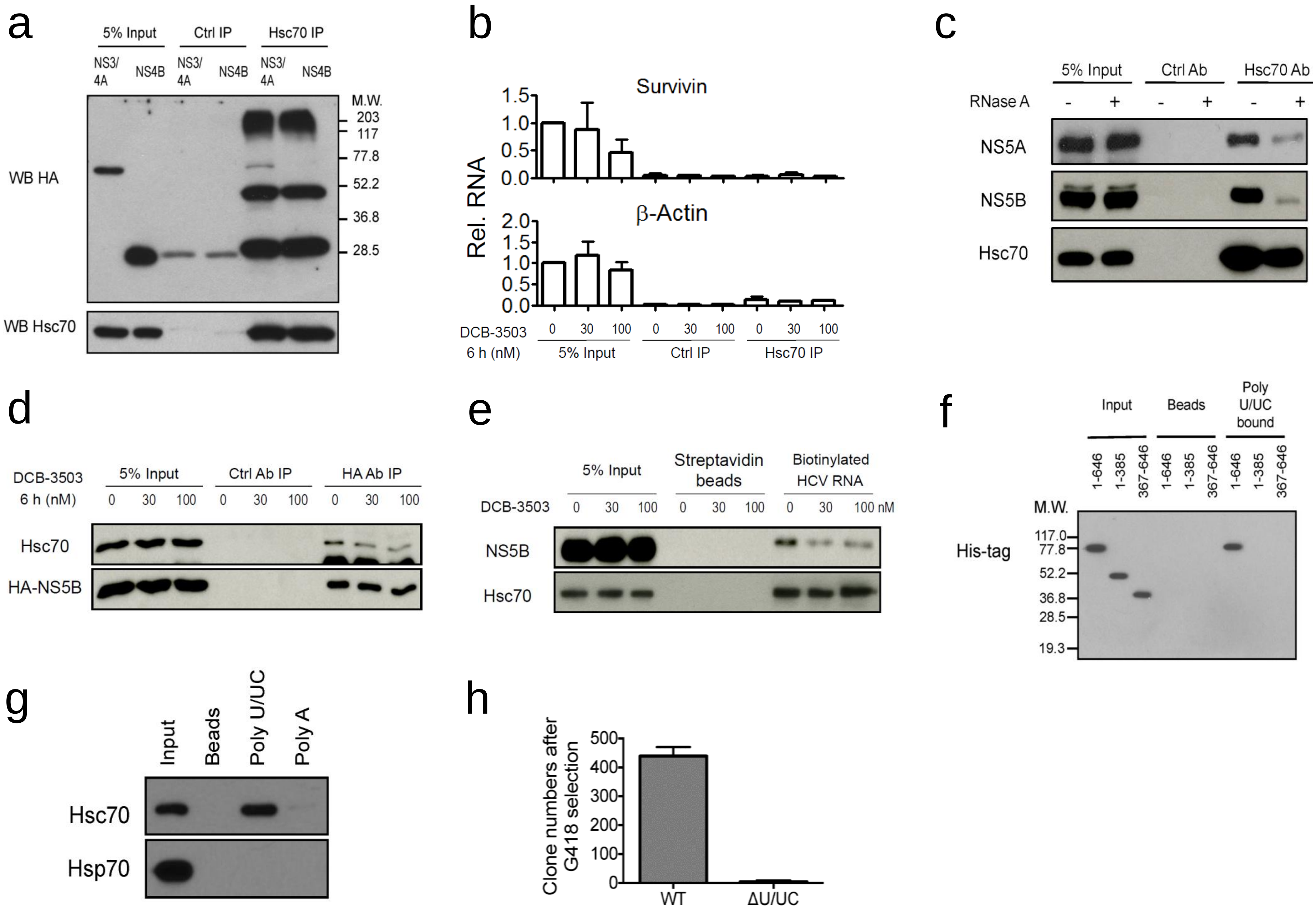

a
5% Input
Ctrl IP
Hsc70 IP
NS3/4A
NS4B
M.W.
203
117
77.8
52.2
36.8
28.5
WB HA
WB Hsc70
b
Survivin
β-Actin
Rel. RNA
1.5
1.0
0.5
0.0
2.0
DCB-3503 6 h (nM)
0
30
100
5% Input
Ctrl IP
Hsc70 IP
c
5% Input
Ctrl Ab
Hsc70 Ab
RNase A
-
+
NS5A
NS5B
Hsc70
d
DCB-3503 6 h (nM)
5% Input
Ctrl Ab IP
HA Ab IP
Hsc70
HA-NS5B
e
5% Input
Streptavidin beads
Biotinylated HCV RNA
DCB-3503
100 nM
NS5B
Hsc70
f
Input
Beads
Poly U/UC bound
1-646
1-385
367-646
M.W.
His-tag
117.0
77.8
52.2
36.8
28.5
19.3
g
Input
Beads
Poly U/UC
Poly A
Hsc70
Hsp70
h
Clone numbers after G418 selection
500
400
300
200
100
0
WT
ΔU/UC

Figure S4

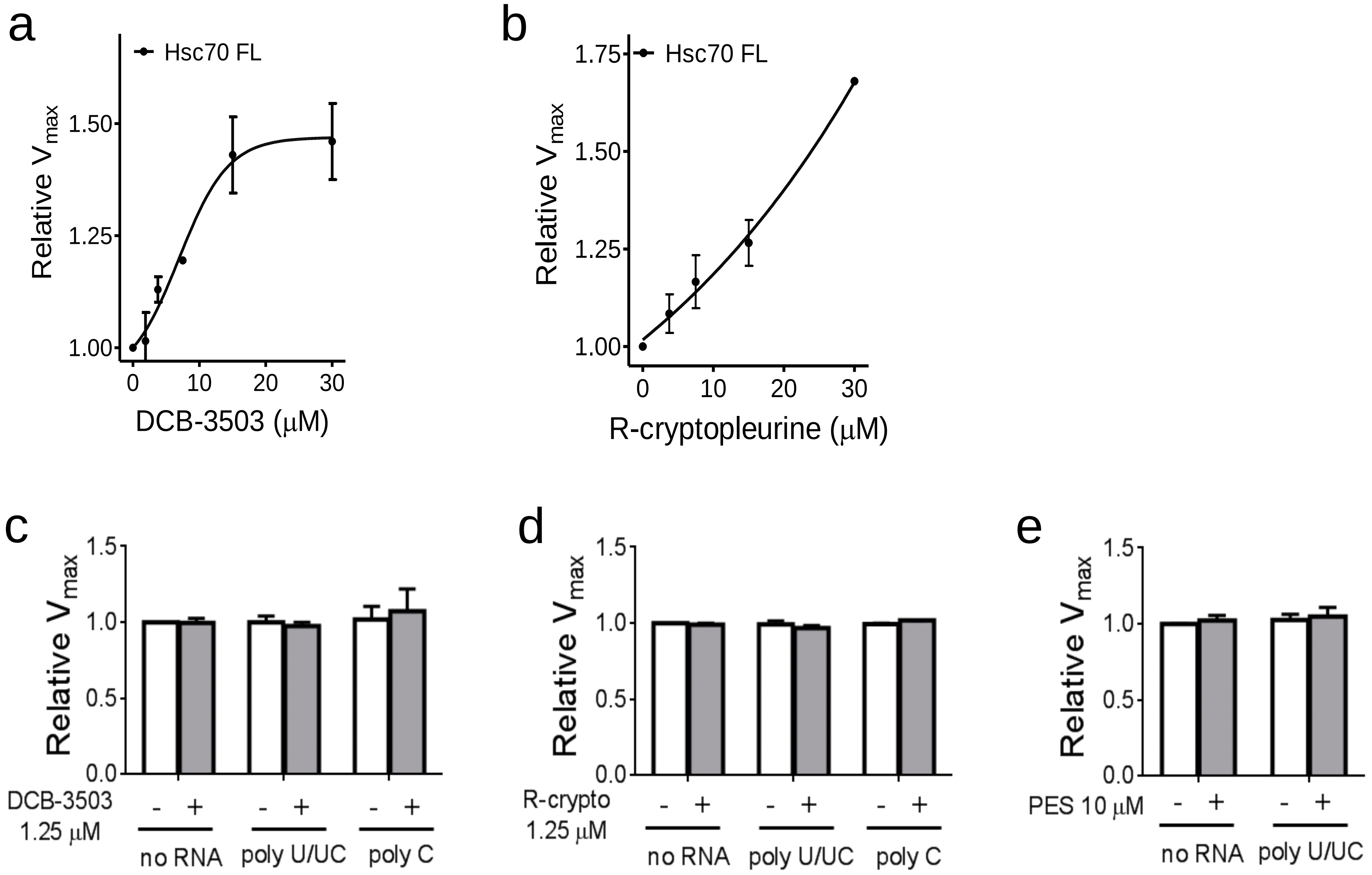
a
Hsc70 FL
Relative V_{max}
1.50
1.25
1.00
0
10
20
30
DCB-3503 (μM)
b
Hsc70 FL
Relative V_{max}
1.75
1.50
1.25
1.00
0
10
20
30
R-cryptopleurine (μM)
c
Relative V_{max}
1.5
1.0
0.5
0.0
DCB-3503 1.25 μM
- + - + - +
no RNA
poly U/UC
poly C
d
Relative V_{max}
1.5
1.0
0.5
0.0
R-crypto 1.25 μM
- + - + - +
no RNA
poly U/UC
poly C
e
Relative V_{max}
1.5
1.0
0.5
0.0
PES 10 μM
- + - +
no RNA
poly U/UC
